# Impacts of local adaptation of forest trees on associations with herbivorous insects: implications for adaptive forest management

**DOI:** 10.1111/eva.12329

**Published:** 2015-10-13

**Authors:** Frazer H Sinclair, Graham N Stone, James A Nicholls, Stephen Cavers, Melanie Gibbs, Philip Butterill, Stefanie Wagner, Alexis Ducousso, Sophie Gerber, Rémy J Petit, Antoine Kremer, Karsten Schönrogge

**Affiliations:** 1 Institute of Evolutionary Biology, University of EdinburghEdinburgh, UK; 2 Centre for Ecology and HydrologyWallingford, UK; 3 Centre for Ecology and HydrologyEdinburgh, UK; 4 Faculty of Science, Biology Center, The Czech Academy of Sciences, University of South BohemiaČeské Budějovice, Czech Republic; 5 INRA, UMR 1202 BIOGECOCestas, France; 6 UMR 1202 BIOGECO, University of BordeauxTalence, France

**Keywords:** adaptive forest management, climate matching, gallwasp, local adaptation, plant–insect interactions, population nonindependence, provenance trials, *Quercus petraea*

## Abstract

Disruption of species interactions is a key issue in climate change biology. Interactions involving forest trees may be particularly vulnerable due to evolutionary rate limitations imposed by long generation times. One mitigation strategy for such impacts is Climate matching – the augmentation of local native tree populations by input from nonlocal populations currently experiencing predicted future climates. This strategy is controversial because of potential cascading impacts on locally adapted animal communities. We explored these impacts using abundance data for local native gallwasp herbivores sampled from 20 provenances of sessile oak (*Quercus petraea*) planted in a common garden trial. We hypothesized that non-native provenances would show (i) declining growth performance with increasing distance between provenance origin and trial site, and (ii) phenological differences to local oaks that increased with latitudinal differences between origin and trial site. Under a local adaptation hypothesis, we predicted declining gallwasp abundance with increasing phenological mismatch between native and climate-matched trees. Both hypotheses for oaks were supported. Provenance explained significant variation in gallwasp abundance, but no gall type showed the relationship between abundance and phenological mismatch predicted by a local adaptation hypothesis. Our results show that climate matching would have complex and variable impacts on oak gall communities.

## Introduction

Ecosystems across the world are experiencing profound climate change (IPCC, 2013), driving population responses whose effects can influence community structure and function (Walther et al. 2002; Parmesan and Yohe 2003). Forest ecosystems are expected to be particularly sensitive to pressures imposed by climate because the long generation times of trees result in low rates of adaptation (Lindner et al. 2010). Furthermore, because forest trees are foundation (and also often keystone) species for associated communities of plants, animals, fungi and microbes (Kennedy and Southwood 1984; McEwan et al. 2011; Lindenmayer et al. 2014), effects on them are expected to cascade through associated trophic levels (Frelich et al. 2012). Consequently, there is considerable interest in developing adaptive forest management strategies to preserve ecosystem productivity and services in the face of climate change (Spittlehouse and Stewart 2003; Broadmeadow et al. 2005; Aitken et al. 2008; Bower and Aitken 2008; Bolte et al. 2009). Assisted migration, in the form of climate matching, has emerged as one potential strategy facilitating forest adaptation to predicted future climates (Hoegh-Guldberg et al. 2008; Dawson et al. 2011), particularly in commercial forestry where potential for *in situ* adaptation may be limited (Broadmeadow et al. 2005; but see Cavers and Cottrell 2014). Climate matching is based on the premise that natural forest stands are locally adapted to current local climates (Savolainen et al. 2007, 2013; Alberto et al. 2013a), and for a given species and planting location involves the following steps: (i) identify climate variables that predict performance for the target tree species across its range; (ii) use regional climate models to predict future climates at the proposed planting site; (iii) identify locations within the species range that currently experience the predicted conditions; (iv) plant seed from tree populations in the identified locations (termed provenances in forestry) in the expectation that resulting trees will be well matched to future climates, and contribute adaptive variation that accelerates subsequent local adaptation (Broadmeadow et al. 2005).

As remediation strategies, climate matching and other forms of assisted migration remain subjects of vigorous debate (Dawson et al. 2011; Lunt et al. 2013; Cavers and Cottrell 2014). Forests are biologically diverse ecosystems, and the value of climate matching, or any other strategy that modifies the genetic, physical or spatial structure of tree populations, must be considered not only in terms of its effectiveness in promoting forest adaptation and resilience, but also its impacts on local associated biodiversity. Community genetic research has shown that the organisms associated with forest stands reflect the genotypes and genetic diversity of the host trees (Dungey et al. 2000; Wimp et al. 2005; Whitham et al. 2012), and a range of studies have shown adaptation of plants to local herbivores and *vice versa* (Sork et al. 1993; Hanks and Denno 1994; Egan and Ott 2007; Tack and Roslin 2010; Bernhardsson et al. 2013). As such, the introduction of new tree genotypes as part of a climate matching strategy may drive changes in the identity, abundance and genetics of associated species (Lefevre et al. 2014). However, it is difficult to forecast the direction and magnitude of such changes. While some studies predict increased abundance of herbivores on non-native tree provenances (Bernhardsson et al. 2013), others suggest the opposite through local herbivore adaptation to plant phenology (Van Asch and Visser 2007; Visser 2008; Pearse and Karban 2013). The key issues are the extent to which host plant provenances vary in phenotypic traits relevant to herbivore success, and the magnitude of herbivore population responses to such variation.

Here we explore the community effects of climate matching by quantifying the impact of provenance on (i) the phenotype (growth performance and phenology) of an important and widespread European oak, *Quercus petraea*, and (ii) the abundance of associated insect herbivores. Our approach exploits an established INRA forestry trial at La Petite Charnie in Northwest France, where 96 provenances of *Q. petraea* grow surrounded by natural oak forest and are exposed to native herbivores. Our study focuses on oak cynipid gallwasps (Hymenoptera; Cynipidae), specialized herbivores whose larvae develop in galls on their oak hosts (Schönrogge et al. 2000; Stone and Schönrogge 2003; Harper et al. 2004). Gall induction requires access to host oak tissues at the correct developmental stage (Harper et al. 2004), and oak cynipids are highly sensitive to host plant phenology (Askew 1962; Crawley and Akhteruzzaman 1988). These aspects of gallwasp biology, coupled with a partly parthenogenetic lifecycle, are associated with local adaptation to specific host plant populations (Mopper et al. 2000; Mopper 2005; Egan and Ott 2007; Tack et al. 2010) and individuals (Egan and Ott 2007) and make gallwasps an appropriate sentinel taxon for exploration of the potential effects of climate matching.

We expect the performance of oak provenances to correlate positively with the extent to which they are adapted to prevailing climatic conditions (Broadmeadow et al. 2005; Lefevre et al. 2014). This hypothesis predicts that performance of locally native provenances will decline over time – and recent rapid climate change may mean that populations of long-lived trees are already somewhat mismatched to the conditions in which they grow. The hypothesis also predicts that provenances selected to match conditions furthest in the future should be least well matched to current conditions and so perform least well in the present. We test this hypothesis using multiple performance measures for 20 provenances planted at the La Petite Charnie provenance trial, selected to maximize the available range in source latitude and longitude as well as the mismatch in climate between the provenance trial and the provenance origin. We then test two contrasting hypotheses linking herbivorous insect abundance to observed variation in performance among oak provenances. The plant stress hypothesis predicts that herbivores should show improved performance and abundance on physiologically stressed plants, due to higher levels of mobilized nitrogen in stressed plant tissues and an associated increase in resource quality for herbivores (White 1969, 1974, 2009). In contrast, the plant vigour hypothesis (Price 1991; Cornelissen et al. 2008) predicts increased herbivore performance and abundance on more vigorous plants or plant organs (those with high growth rates or large ultimate size relative to the population mean) through elevated nutrient content and reduced concentrations of defensive compounds. Here we test both hypotheses using abundance data across provenances for 20 cynipid gall types.

Phenological matching of key biological events is crucial for many interactions between forest trees and associated organisms. For example, synchronization of breeding with high plant resource availability (budburst for leaf-eating herbivores) is central to reproductive success in herbivores and associated trophic levels. We thus expect associated organisms to show local adaptation to host plant phenology (Ducousso et al. 1996; Yukawa 2000; Van Asch and Visser 2007; Van Asch et al. 2012; Alberto et al. 2013b). Many plants show latitudinal clines in phenological events, such as the timing of budburst, whose onset is often determined by photoperiodic cues (Savolainen et al. 2007; Aitken et al. 2008) or the interaction between photoperiod and temperature (Laube et al. 2014). This is relevant to climate matching because, in a warming world, it often involves introducing plants to higher latitudes from lower latitudes. As a result, phenological mismatches with local herbivores might occur by disrupting any adaptation to the local combination of thermal and photoperiod cues, with associated impacts on the fitness of herbivores and trophically linked taxa such as natural enemies (Visser 2008; Thomson et al. 2010; Kerstes and De Jong 2011; Van Asch et al. 2012). Here we quantify the phenotypic mismatch in budburst date between the same 20 provenances and assess the impact of phenological mismatch on cynipid gall abundance. Specifically, we test the local adaptation hypothesis, that is that herbivore abundance will decline with increasing phenological difference between introduced and locally native oak provenances. We distinguish phenological effects from other potential differences between provenances (e.g., variation in chemical defences) by incorporating provenance phenology into our analyses.

Our analytical approach recognizes that tree populations are linked by shared common ancestry and gene flow and hence are unlikely to represent statistically independent replicates (Stone et al. 2011). Here we investigate the impact of potential nonindependence by comparing the results of analyses that incorporate the expected variance–covariance among populations in a generalized linear mixed model framework (Hadfield and Nakagawa 2010).

## Materials and methods

### Provenance trial design

**Figure 1:**
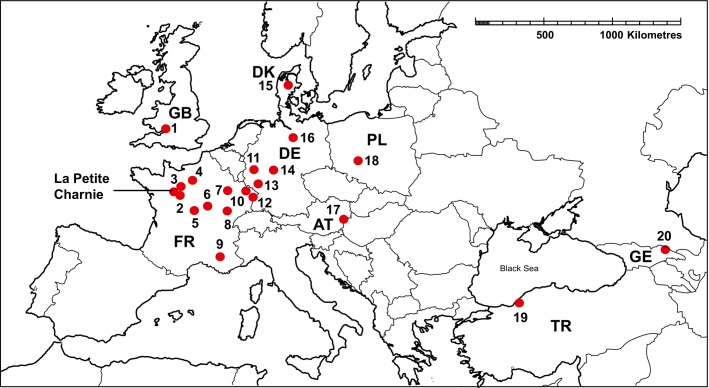
Location of the Petite Charnie provenance trials and the 20 selected study provenances. Sites are identified by number in Table1. Countries contributing provenances are indicated by their two letter ISO codes.

**Figure 2:**
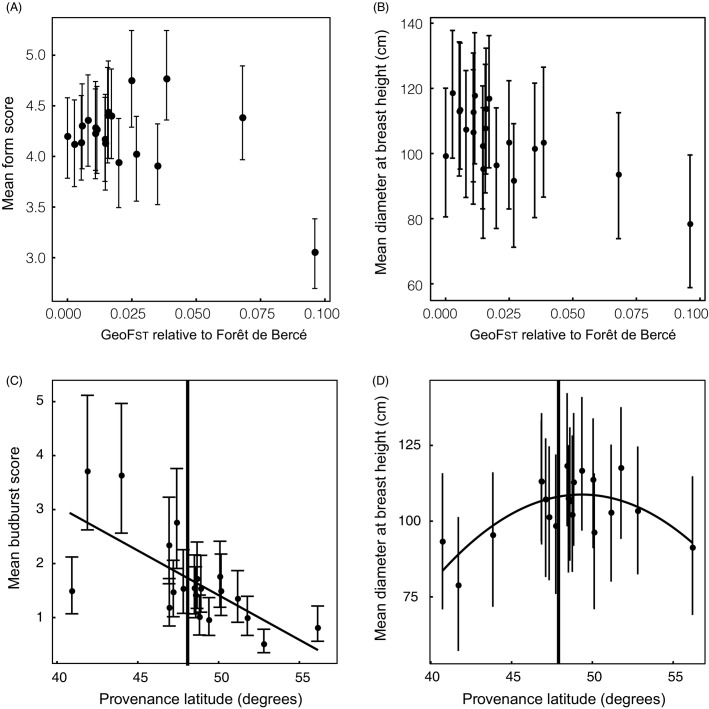
Between-provenance variation in three phenotypic variables (A) *Form*, in which higher values indicate healthier trees; (B) and (D) *Diameter at breast height* (*DBH*), in which higher values indicate more vigorous growth; and (C) *Budburst* date, in which higher values indicate earlier budburst in the spring. In (A) and (B), the position of provenances on the *x-axis* relates to their genetic differentiation (estimated as *GeoF*_ST_) from the provenance closest to the provenance trial site (Forêt de Bercé). In (C) and (D), their position relates to the latitude of their site of origin with the identity of Forêt de Bercé shown. Modelling was conducted in the MCMCglmm r package, and response variables were modelled with provenance fitted as a fixed effect and provenance, soil zone, plot and tree fitted as random effects. Models of (A) and (C) were fitted with a Poisson distribution, and (B) and (D) with a Gaussian distribution. Priors, burn-in, number of iterations and sampling frequency follow that described previously for the modelling of provenance effects on gall abundance. Vertical bars represent 95% confidence intervals for each mean. MCMC, Markov Chain Monte Carlo.

Provenance effects were explored using a large experimental trial of *Q. petraea* at La Petite Charnie in Northwest France (Fig. 1; 48.086° north, 0.168° west; details on the planting and layout of the trial are provided in Data S1). We selected 20 provenances whose origins span 15° of latitude and 34° of longitude, incorporating the greatest available geographic separation and source environmental variation (Fig. 1, Table S1 in Data S1), and included the closest provenance to the trial site (Forêt de Bercé, approximately 50 km from La Petite Charnie). Each sampled provenance is planted in multiple replicate plots of 24 trees distributed across five soil zones (see Data S1). We selected two plots per soil zone per provenance, giving a total of 200 study plots containing 4800 trees. Previous INRA surveys provided data for each tree for the following phenotypic traits (see Data S1 for illustrations of character states):

1. *Budburst* – spring budburst phenology was scored on a 0–5 ordinal scale, where 0 is a dormant bud and 5 is a fully open bud, in spring 1995 (this closely parallels that used in other studies on oak (Crawley and Akhteruzzaman 1988; Tack et al. 2010). A higher score on the survey date indicates earlier budburst. Although oak phenology is not known to show strong *G* × *E* interactions across years (Alberto et al. 2011), we nevertheless repeated phenology scoring in 2009 for 582 trees in a subset of eight provenances. Phenology scores for the same tree in 1995 and 2009 were significantly and positively correlated (see Data S4). We thus regard using the 1995 data for all 20 provenances as unlikely to produce misleading or contrary results.
2. *Diameter at breast height* (*DBH*) – tree diameter at a height of 1.3 m, measured during winter 2001–2002. A higher value is associated with more rapid growth and higher plant vigour.
3. *Form* – a measure based on tree shape, recorded on an ordinal scale from 1 to 10 during winter 2001–2002. Stressed trees with bushy, irregular growth and poor apical dominance receive a low score, while healthy trees with straight stems, even branching and strong apical dominance score highly. These phenotypic measures showed substantial variation among provenances (discussed in more detail below), providing the basis for examination of impacts of phenological matching (*Budburst*) and performance (*DBH*, *Form*) on herbivore abundance (Table1, Fig. 2).

**Table 1:** Summary of the 20 studied provenances of *Quercus petraea,* ordered by longitude from west to east showing their three digit INRA provenance codes, provenance origin (site name and country), latitude and longitude in decimal degrees, altitude (Alt), the availability of genotypic data and the mean values across all trees of each provenance for the phenotypic traits *Budburst, Diameter at breast height* (*DBH*) and *Form*

Site number in Fig. 1	Code	Provenance name	Country	Long (DD)	Lat (DD)	Alt (m)	Genotypic data	Mean *Budburst*	Mean *DBH*	Mean *Form*
1	185	Blakeney	UK	−2.5	51.78	76	No	1.08	120	4.24
**2**	**217**	**Bercé**	**France**	**0.39**	**47.81**	**155**	**Yes**	**1.59**	**98**	**4.2**
3	237	Réno Valdieu	France	0.67	48.5	230	Yes	1.65	116	4.14
4	210	Saint Germain	France	2.08	48.9	60	Yes	1.71	113	4.13
5	194	Soudrain	France	2.38	46.95	178	Yes	1.24	110	4.33
6	211	Prémery	France	3.6	47.2	300	Yes	1.55	106	4.34
7	201	La Haie Renaut	France	4.95	48.67	180	Yes	1.77	109	4.24
8	245	Étangs	France	4.96	46.93	200	Yes	2.4	113	4.19
9	233	Vachères	France	5.63	43.98	650	Yes	3.69	94	4.15
10	230	Romersberg	France	6.73	48.82	220	No	1.06	101	4.17
11	250	Cochem	Germany	7.05	50.08	400	Yes	1.86	112	4.43
12	225	Still	France	7.25	48.58	688	Yes	1.55	105	4.56
13	252	Johanneskreuz	Germany	7.83	49.4	460	Yes	1.03	116	4.42
14	257	Wolfgang	Germany	9.05	50.15	160	Yes	1.61	96	3.92
15	181	Horbylunde	Denmark	9.41	56.13	80	Yes	0.88	89	3.99
16	255	Spakensehl	Germany	10.6	52.8	115	Yes	0.54	102	4.74
17	248	Kloster-marienberg	Austria	16.57	47.41	310	Yes	3.23	103	3.9
18	179	Sycow	Poland	17.93	51.18	210	Yes	1.42	104	4.76
19	249	Bolu	Turkey	31.67	40.92	1200	Yes	1.58	94	4.39
20	184	Telavi	Georgia	45.47	41.88	700	No	3.79	77	3.06

Values for the provenance closest to the trial site, Forêt de Bercé, are shown in bold.

### Herbivore sampling

The cynipid species surveyed in this study all have broad geographic distributions; like *Q. petraea* and closely related oaks, they span the Western Palaearctic from the Iberian Peninsula eastwards to Iran and the Caucasus. Cynipid gall abundance was sampled across the 20 selected provenances in 2008 and 2009. Gallwasps have a spring (sexual) and autumn (asexual) gall generation each year, and both were sampled, giving four surveys in total. For each of the 200 study plots, we sampled galls from 12 of the 24 available trees, a total of 2400 trees. To minimize edge effects, surveyed trees comprised the two internal rows of six trees within each plot. During each survey, using a pole pruner giving us approximately 4 m reach we selected 10 twigs on each tree, each consisting of the last 2 years’ growth identified using ring scars on the bark. For each twig, we surveyed the galls on all parts (buds, leaves, stem, catkins, acorns; there are no cynipid galls on female flowers in Europe) of its terminal shoot (a module of woody growth from the previous year carrying the new leaves). Galls were identified to species and generation in the field using a morphological key (Redfern and Shirley 2002) and our own extensive field experience with this group (Stone et al. 2002; Csóka et al. 2005).

### Statistical analysis

#### Controlling for statistical nonindependence of provenances

One way to address potential statistical nonindependence of populations within species is to incorporate expected similarities among populations (due to shared common ancestry and/or gene flow) as one or more variance–covariance matrices in the random effect structure of a generalized linear mixed model (Stone et al. 2011). Covariance matrices for nonindependence arising through each of migration and common ancestry can be generated for small numbers of populations using population genomic data (e.g., Hearn et al. 2014). However, for larger numbers of populations (as here), and where more limited genotypic data are available, an alternative is to use a measure of pairwise genetic differentiation between populations such as *F*_ST_ (Weir and Cockerham 1984) as an estimate of their expected covariance (Stone et al. 2011). Where genetic differentiation between populations correlates strongly with geographic distance, variance–covariance can be estimated as a function of geographic separation between populations.

We used data from 10 putatively neutral microsatellite markers (listed in Data S2; Guichoux et al. 2011) available for 17 of our 20 study provenances (Table1) to estimate pairwise *F*_ST_ as implemented in fstat version 2.8.3.2 (Weir and Cockerham 1984; Goudet 1995). For comments on the impact of using alternative measures of genetic differentiation related to *F*_ST_, see Data S2. To allow us to analyse data for all 20 study provenances, we used the highly significant relationship between *F*_ST_ and geographic distance for the 17 provenances (Mantel test in the *adegenet* r package, *P *=* *0.01 with 999 iterations) to estimate a pseudo-*F*_ST_ for the full set of 20 provenances, referred to hereafter as *GeoF*_ST_ (full details are given in Data S2). Variance–covariances between provenances were then estimated as either 1−*F*_ST_ or 1−*GeoF*_ST_, such that expected covariance was greatest between provenances with lowest differentiation.

To explore the consequences of using alternative approaches to control for provenance nonindependence, all of the analyses described below were conducted using the following five dataset/covariance matrix combinations: (i) the subset of 17 provenances for which genotypic data were available, using an identity matrix as the provenance covariance matrix. This identity model assumes provenances to be statistically entirely independent (i.e., 0 covariance between all populations); (ii) the same 17 provenances, using a covariance matrix estimated as 1−*F*_ST_; (iii) the same 17 provenances, using a covariance matrix estimated as 1−*GeoF*_ST_; (iv) the full set of 20 provenances using an identity model; and (v) the full set of 20 provenances where covariance is estimated as 1−*GeoF*_ST_. A detailed discussion of this approach to addressing population nonindependence, and values used in the covariance matrices, are provided in the Data S2.

#### Statistical modelling of provenance effects on gall abundance

To investigate the influence of tree provenance on the abundance of each individual gall type, we combined the data for the two survey years and used generalized linear mixed models (GLMMs) to partition the variance of gall counts per shoot into the effects of year (as a fixed effect with two levels) and provenance, soil zone, plot and tree (all as random effects). When applying an identity model for the relationship between provenances, the effect of provenance was represented by a single random effect with a unique independent level for each provenance. In models that incorporated the relationship between provenances, provenance co-variance was represented by two random effects. One incorporated the specified variance–covariance matrix (1−*F*_ST_ or 1−*GeoF*_ST_) to account for provenance nonindependence, while the second incorporated an identity matrix to capture the ‘nugget effect’ (Matheron 1963), in which variation in the response variable exceeds that expected through correlation between levels of the effect. Models were fitted in a Bayesian Markov Chain Monte Carlo (MCMC) framework using the package MCMCglmm version 2.16 (Hadfield 2010) in r version 2.15.1. For fixed effects, prior settings assumed a normal distribution with a mean (*µ*) of 0 and a variance (*V*) of 10^8^. For random effect variances, scaled *F*_{1,1} priors (with scale 1000) were used (Gelman 2006) except for the residual variance for which an inverse-gamma prior was used with shape and scale equal to 0.001. Models were run for 500 000 iterations with a burn-in of 50 000 and parameter sampling every 450 iterations. As the response variable was count data and usually contained many zeros, a Poisson error distribution was applied. Our incorporation of *year* as a fixed effect allowed for variation in abundance between years, while estimating the relationship between gall abundance and tree phenotypic traits across both years. The proportion of variance explained by the *year* fixed effect was estimated as the marginal *R*^2^ of the model (RsqM), following Nakagawa and Schielzeth (2013) (r code for deriving RsqM from MCMCglmm objects is provided in Data S3). The variance component of each random effect was calculated by dividing its estimated variance parameter by the sum of all the random effect variance parameters, including the residual UNIT term – a random effect with a distinct level for each data unit that is fitted automatically for Poisson distributed models in the MCMCglmm package to account for overdispersion – and then multiplying each by the proportion of variance not explained by the fixed effect (i.e., 1−RsqM). We addressed the effects of multiple tests on false discovery rates – the expected proportion of false discoveries among the rejected hypotheses – following Benjamini and Hochberg (1995) and Benjamini and Yekutieli (2001). The false discovery rate is a less stringent condition than the family-wise error rate, so this approach is more powerful than alternatives such as Bonferroni (Nakagawa 2004).

### Effect of tree phenotype

To investigate whether tree phenotypic traits were good predictors of variation in gall abundance, GLMMs were used to model counts of individual gall types with *DBH* (growth), *Form* (tree health) or *Budburst* (spring phenology) as fixed effects. While the influences of tree growth and health on gall abundance are expected to be linear, the local adaptation hypothesis for herbivores predicts that the influence of phenology will be curvilinear, with lower gall abundance on provenances that show budburst either before or after local oaks. The fixed effect *Budburst* was therefore fitted as both untransformed and squared terms. To account for potential differences in abundance levels between the two study years, a factor *year* was fitted as a fixed effect.

For each gall type, counts per shoot were initially modelled with *year* and either *Form*, *DBH*, *Budburst* or both *Budburst* and *Budburst*^2^ as fixed effects. The severity of co-linearity between these variables was assessed by their variance inflation factors (VIFs) using the r package *HH* version 2.3-23 (Heiberger 2013). To assess the importance of interactions between tree phenotypic traits, a model containing the fixed main effects *Form*, *DBH*, *Budburst*, and their three pairwise interactions was fitted for each gall type. All models were fitted in the MCMCglmm package with the random effect structures and prior specifications described above. The probability that the posterior distribution of the fixed effect coefficients did not include zero (pMCMC) was used to assess the predictive capacity of the fixed effects.

## Results

### Phenotypic variation among provenances

If provenances are locally adapted, and divergence in selection history between provenance origins and the trial site increases with genetic distance, then we expect more genetically divergent provenances to show higher stress (measured using *Form*) and lower vigour (measured using *DBH*). *Form* shows no linear relationship, but increases in variance in more divergent provenances (Fig. 2A). Only the most genetically divergent provenance, from Telavi in Georgia, showed a markedly lower *Form* score than other provenances. Plant vigour, measured using *DBH*, does decrease with increasing genetic divergence (Fig. 2B), consistent with local adaptation. Budburst phenology shows a strong relationship with latitude (Fig. 2C), with more southerly provenances showing earlier budburst: by the time some southern provenance trees have open leaves (scores 4 and 5), northern provenances have yet to burst their buds (scores 0 and 1). We thus expect phenological mismatch with native oaks to correlate with differences in latitude between provenance origins and the trial site.

### Herbivore abundance

We recorded approximately 725 000 galls of 20 gall types (seven sexual generation and 13 asexual generation) during the four survey seasons, representing 15 different gallwasp species (Table2). No acorn galls were recorded. Total counts of individual gall types varied over five orders of magnitude, from just 4 (asexual generation *Cynips longiventris*) to over 330 000 (asexual generation *Neuroterus anthracinus*). Gall abundance differed significantly between years for all gall types except *Andricus solitarius*, with five types exhibiting >10-fold differences (Table2) and without any consistent pattern between years or seasons. Six of the 20 gall types occurred on <1% of shoots in both survey years and were excluded from further analysis.

**Table 2:** Prevalence, incidence and between-year variation in the abundance of the 20 gall types recorded at La Petite Charnie. Columns show, for each survey year, the total number of each gall type, the mean number of galls per shoot and incidence – the proportion of shoots bearing galls, and the ratio of mean galls per shoot between 2008 and 2009

Gall type (species and generation)	2008			2009			Ratio of mean galls/shoot 2008:2009
	Total galls	Mean galls/shoot	Incidence	Total galls	Mean galls/shoot	Incidence	
Spring sexual generation surveys							
*Andricus inflator*	52	0.0022	0.0018	0	0	0	–
*Andricus testaceipes*	973	0.041	0.0025	2291	0.095	0.067	1:2.3
*Biorhiza pallid*a	13	0.00054	0.00046	10	0.00042	0.00042	1.3:1
*Neuroterus albipes*	822	0.034	0.03	5828	0.24	0.16	1:8
*Neuroterus anthracinus*	14 278	0.59	0.35	7411	0.31	0.23	1.9:1
*Neuroterus numismalis*	1154	0.048	0.039	17 486	0.73	0.29	1:15
*Neuroterus quercusbaccarum*	5808	0.24	0.15	7834	0.33	0.2	1:1.4
Autumn asexual generation surveys							
*Andricus callidoma*	4	0.00021	0.00021	45	0.0019	0.0018	1:9
*Andricus fecundatrix*	334	0.017	0.013	1103	0.046	0.029	1:2.5
*Andricus glandulae*	83	0.0043	0.0037	335	0.014	0.012	1:2.3
*Andricus inflator*	44	0.0023	0.002	3	0.00013	0.00013	19:1
*Andricus kollari*	2	0.0001	0.0001	43	0.0018	0.0012	1:18
*Andricus solitarius*	528	0.028	0.026	726	0.03	0.028	1:1
*Cynips divisa*	1901	0.099	0.057	184	0.0077	0.0057	13:1
*Cynips longiventris*	1	0.000052	0.000052	3	0.00013	0.00013	1:2.5
*Cynips quercusfolii*	197	0.01	0.0085	456	0.019	0.013	1:2
*N. albipes*	45 771	2.38	0.51	27 531	1.15	0.32	2.1:1
*N. anthracinus*	313 507	16.33	0.93	23 820	0.99	0.29	16:1
*N. numismalis*	12 194	0.64	0.037	12 124	0.51	0.028	1.2:1
*N. quercusbaccarum*	123 708	6.44	0.56	94 830	3.95	0.49	1.6:1

### Oak provenance effects on gall abundance

**Figure 3:**
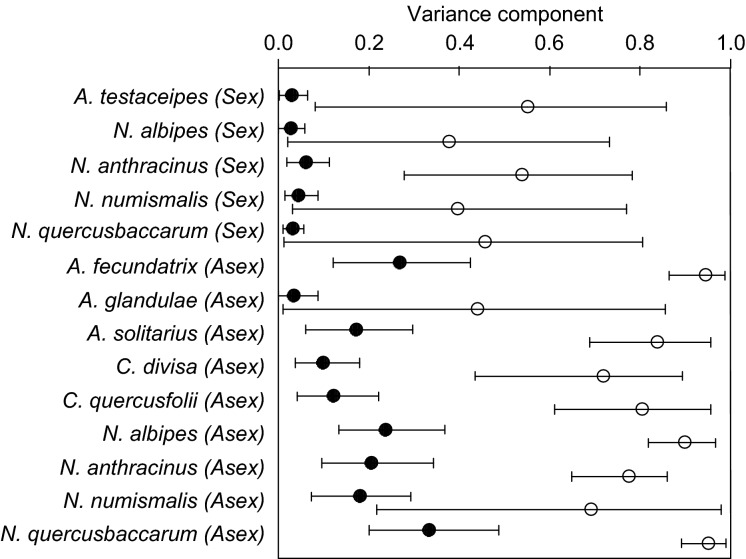
The proportion of variation in gall abundance attributed to the effect of provenance for the 20-provenance dataset that either do not (black circles) account for population nonindependence (i.e., identity models) or incorporate a 1−*GeoF*_ST_ variance–covariance matrix (open circles). Vertical bars represent 95% confidence intervals for each mean.

The percentage of variance in gall abundance per shoot explained by differences between provenances (expressed as the mean variance component, ranging between 0% and 100%) varied substantially between the statistical models used. In identity models of the 20 provenance dataset (which assume provenances to be fully independent statistically), the percentage of variance in gall abundance explained by provenance ranged from 3% (in sexual generation *Neuroterus albipes*) to 33% (in asexual generation *Neuroterus quercusbaccarum*) and was >10% for seven of the 14 gall types (Fig. 3). Because provenances are related, the identity model is unlikely to be appropriate. All models incorporating provenance covariance gave higher estimates of the variance component for provenance effects (see Data S2 for comparison of results across the alternative datasets and models). Incorporation of provenance covariance using the 1−*GeoF*_ST_ covariance matrix for the same 20 provenance dataset attributes between 38% (sexual generation *N. albipes* and 95% (asexual generation *N. quercusbaccarum*) of variance in gall abundance to provenance effects (Fig. 3). The identity models should thus be viewed as providing artificially low estimates of the variation in gall abundance attributable to differences between provenances (Table S2.2, Figure S2.1 in Data S2). On this basis, host tree provenance is an important predictor of the abundance of many gallers, explaining at least 10%, and as much as 95% of the variation in abundance of seven of the 14 gall types tested. Without more knowledge of the form of variance–covariance relationship, it is not possible to give a correct estimate. Importantly for what follows, analysis of fixed effects was not influenced by our choice of variance–covariance model.

### Tree phenotypic traits as predictors of variation in gall abundance

Significant relationships between gall abundance per shoot and at least one of the provenance phenotypic traits were identified for all but three of the 14 gall types tested (Table3). The signs and strengths of relationships were stable across the various datasets and provenance co-variance structures (see Data S2 for full comparison of results), and here, we therefore focus on the results of the 1−*GeoF*_ST_ co-variance matrix models for the 20-provenance dataset, identifying where they differ from the identity models for the same dataset (Table3). The phenotypic variables *Form, DBH* and *Budburst* were not confounded, with low VIFs (1.05, 1.02 and 1.02, respectively). Because our analyses yielded only one significant interaction term (*Budburst*:*Form* for sexual generation galls of *Neuroterus numismalis*; mean coefficient = 0.031, pMCMC = 0.014), hereafter we consider each trait in isolation.

**Table 3:** Summary of GLMMs for tree phenotypic predictors, showing the significance and sign of those fixed effect coefficients that differed significantly from zero. Significance was assessed using pMCMC. Where the result of the identity models differed from the 1−*GeoF*_ST_ models, the identity result follows the 1−*GeoF*_ST_ result

Gallwasp species (generation)	Year	*Form*	*DBH*	*Budburst*	*Budburst* ^2^	*Budburst: DBH*	*Budburst: Form*	*Form: DBH*
*Andricus testaceipes* (sex)	***	NS	+**/+*	NS	NS	NS	NS	NS
*Neuroterus albipes* (sex)	***	NS	NS	NS	NS	NS	NS	NS
*Neuroterus anthracinus* (sex)	***	NS	NS	+*	NS	NS	NS	NS
*Neuroterus numismalis* (sex)	***	NS	+**/+***	+***	−*	NS	*	NS
*Neuroterus quercusbaccarum* (sex)	***	NS	NS	+***	NS	NS	NS	NS
*Andricus fecundatrix* (asex)	***	NS	NS	−***	+**/+*	NS	NS	NS
*Andricus glandulae* (asex)	***	NS	NS	−*	NS	NS	NS	NS
*Andricus solitarius* (asex)	NS	NS	NS	NS	NS	NS	NS	NS
*Cynips divisa* (asex)	***	NS	NS	−*	NS	NS	NS	NS
*Cynips quercusfolii* (asex)	***	+*	NS	NS	NS	NS	NS	NS
*N. albipes* (asex)	***	NS	+*	NS	NS	NS	NS	NS
*N. anthracinus* (asex)	***	NS	NS	NS	NS	NS	NS	NS
*N. numismalis* (asex)	***	NS	−*	−***	+*	NS	NS	NS
*N. quercusbaccarum* (asex)	***	NS	−***	−***	+*	NS	NS	NS

DBH, diameter at breast height; MCMC, Markov Chain Monte Carlo.

Significance codes in the table are as follows:

* *P* < 0.05

** *P* < 0.01

*** *P* < 0.001, NS, *P* > 0.05, nonsignificant. Consideration of false-positive discovery rates for multiple testing following Benjamini and Hochberg (1995) and Benjamini and Yekutieli (2001) resulted in adjustment of *P*-values indicated as ^*^ to *P* > 0.05, while all other results indicated as ^**^ or ^***^ in the table below remain significant at *P* < 0.05.

**Figure 4:**
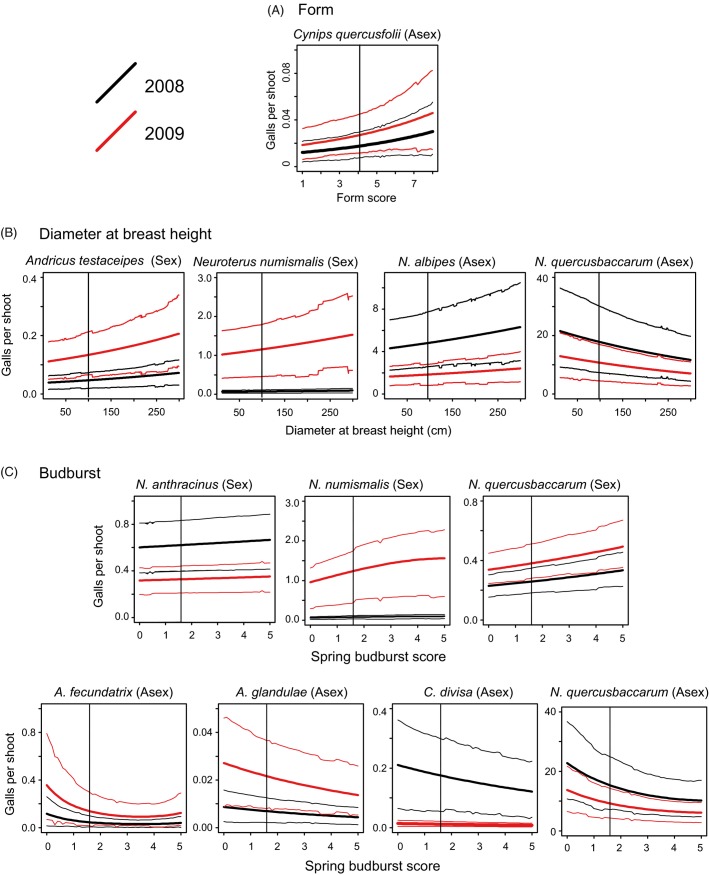
Plots of significant relationships between gall abundance and (A) *Form,* (B) *Diameter at breast height* (*DBH*) and (C) *Budburst*. Higher *Form* scores indicate healthier trees, while higher *Budburst* scores indicate earlier budburst. Distributions of *y* at intervals across the range of a phenotypic predictor (*x*) were derived from the 1000 stored Markov Chain Monte Carlo (MCMC) samples as: *y*_*i*_ = exp(intercept_*i*_ + *x*(mean coefficient_*i*_) + 0.5*x* ∑ random effect variances_*i*_). Plots show the means (bold lines) and 95% confidence intervals from these distributions for each of the two study years. Dotted vertical lines indicate the mean value of the phenotypic trait for the most local tree provenance (Forêt de Bercé). Plots for models of the asexual generation galls of *Neuroterus numismalis* could not be produced in this way due to high random effect variances.

Tree health, as measured by *Form*, significantly predicted the abundance of only one gall type (asexual generation *Cynips quercusfolii*; Table3, Fig. 4A) for which healthier trees supported more galls per shoot, as predicted by the plant vigour hypothesis. Tree vigour, as measured by *DBH*, was a better overall predictor of gall abundance per shoot, with significant relationships for five of the 14 gall types (two sexual and three asexual generations; Table3, Fig. 4B). For the spring sexual generation galls (*Andricus testaceipes* and *N. numismalis*), galls were more abundant on larger trees (Fig. 4B, as predicted by the plant vigour hypothesis). Relationships varied for the three autumn asexual generation gall types: *N. albipes* were more abundant on larger trees (as predicted by the plant vigour hypothesis), while *N. numismalis* and *N. quercusbaccarum* showed the opposite pattern (as predicted by the plant stress hypothesis).

Phenology, as measured by *Budburst*, was the best overall predictor of gall abundance/shoot, with significant correlations for eight gall types (Table3, Fig. 4C). Four gall types showed significantly nonlinear relationships, incorporating a significant *Budburst* squared term. However, no gall type showed peak abundance at an intermediate phenological stage, as expected for the local adaptation hypothesis. Instead, spring sexual generation galls were generally most abundant on provenances showing the earliest budburst (high budburst score), while the summer/autumn asexual generation galls were most abundant on trees showing the latest budburst (low budburst score, Fig. 4C).

## Discussion

Predicting future change in ecosystem functioning requires an understanding of the community impacts of both global environmental change and any associated remediation strategies. Climate change impacts on foundation species are likely to influence the diversity, distribution and abundance of associated taxa – whether these are beneficial ecosystem service providers or potential pests. The challenge for a given system is then to estimate likely consequences of both nonintervention and remediation; and where the impacts of remediation strategies take time to develop, to understand their trajectories through time. All of these aspects apply in particular to climate matching of long-lived foundation trees, which involves introduction of non-native genotypes that take decades to establish and live for centuries. Our analyses shed light on the likely consequences for native herbivores of introducing tree provenances that, although not initially climate-matched, become increasingly so as climate changes, while native provenances become increasingly mismatched and decline in performance. A key aspect of our study is its use of a provenance trial to quantify the consequences of host plant variation in performance and phenology on multiple species in the same guild, addressing a recognized need to quantify the impacts of environmental change at guild and community levels (e.g., Forkner et al. 2008; Van Asch et al. 2012; Ohlberger et al. 2014; Petanidou et al. 2014; Vatka et al. 2014).

### Influences of oak provenance on the gallwasp guild, and incorporation of provenance covariance

Our results show that oak provenances vary significantly in phenotypic traits likely to influence associated herbivores and that this in turn is associated with significant variation in the population density (abundance per oak shoot) of multiple gallwasp species. We show that the form of these relationships is robust across models that assume very different patterns of variance–covariance among oak provenances, from identity models assuming total independence to models that estimate covariance based on population genetic and provenance origin data. Our results indicate that models that treat provenances as statistically independent entities, which we know not to be true, consistently underestimate provenance effects on gall densities relative to models in which covariance is specified based on a measure of genetic differentiation (*F*_ST_ or *GeoF*_ST_). If these models accurately describe provenance co-variance, then the influence of tree provenance on gall abundance is substantially greater (38–95%) than would be inferred by assuming provenances to be statistically independent. While pairwise *F*_ST_ and related measures are consistently significantly larger than 0 (Tables S2.1 and S2.3 in Data S2), their absolute values are small, providing little resolution of the relationship of genetic differentiation and geographic distance (see Data S2 for a comprehensive discussion). It is therefore difficult to estimate a ‘true’ value for the impact of provenance and its explanatory power given the nonindependence of populations (Felsenstein 2002; Stone et al. 2011).

The strong provenance effects we show are consistent with the growing body of community genetics work linking host plant genotypic variation to associated trophic levels (Wimp et al. 2005; Bangert and Whitham 2007). A key challenge in this field is to understand the importance of host genetic effects for herbivore communities relative to other ecological factors (Hersch-Green et al. 2011). Working near the northern limit of pedunculate oak (*Quercus robur*) in Finland, Tack et al. (2010) found spatial effects to be more important than tree genotypic effects in structuring a community of herbivorous insects that included several gallwasps. In reciprocal transplant experiments at local and regional scales, Tack et al. (2010) found no effect of tree genotype for any of the three gall types in their study (asexual and sexual generations of *N. quercusbaccarum* are shared with our study). The contrast between this and the strong host plant provenance effects present at the Petite Charnie trial is likely to reflect contrasting levels of adaptive genetic and phenotypic diversity of the oaks in each study. Although Finnish oak populations show substantial intraspecific genetic diversity (Vakkari et al. 2006), they show substantially less genetic diversity (and we expect also phenotypic diversity) than the much wider geographic array of provenances present at La Petite Charnie (Petit et al. 2002). Our provenance trial study involves a much greater array of genetic diversity than would be expected from a single natural population, a feature shared with the studies of genetically diverse hybrid populations (e.g., Dungey et al. 2000) that often show strong plant genotype effects on associated herbivores (Tack et al. 2010). Generally, the importance of host genetic effects relative to other factors is likely to correlate positively with the adaptive genetic diversity within a tree population present at a site, and we would expect this to be increased in situations where climate matching or other forms of assisted migration are implemented.

### Impacts of between-provenance variation in phenology

The strongest predictor of between-provenance variation in gallwasp density is variation in spring budburst phenology. Phenological changes have long been recognized as a major component of species responses to climate change that can cascade through associated trophic levels. This has been shown, for example, in work on the impact of changes in oak budburst phenology on abundance of associated leaf-feeding moths and breeding responses of insectivorous birds – particularly *Parus major* (Van Asch and Visser 2007; Visser 2008; Van Asch et al. 2012; Vatka et al. 2014). A key question in research on these systems has been the extent to which responses of herbivores, and their natural enemies are the result of phenotypic plasticity and local adaptation.

It is striking that although adaptation to individual trees has been demonstrated in oak–gallwasp interactions (Egan and Ott 2007), none of our gall types showed declining abundance with increasing phenological mismatch between native oaks and planted provenances, rejecting our local adaptation hypothesis. We also found the responses of the spring and autumn generations in the gallwasp lifecycle can be very different. Spring galls were more abundant on early budbursting trees, while autumn generations of the same species (*N. numismalis* and *N. quercusbaccarum*) were more abundant on late budbursting trees. Similar relationships to those we observed were recorded for *Neuroterus* gallwasps in response to local phenological variation in native UK *Q. robur* populations by Askew (1962, 1984) and Crawley and Akhteruzzaman (1988); our results show that the same trends can be extended to the wider phenological variation present in a provenance trial. We hypothesize that this contrast between the spring and autumn generations may exist because later budbursting trees may have higher abundance of phenologically young tissues suitable for gall induction. Annual gallwasp phenology may thus be the result of contrasting selection on the two lifecycle generations, perhaps limiting local phenological adaptation in either generation (Singer and Parmesan 2010). The dynamics of such complex lifecycles are poorly understood, and complex bottom-up impacts may apply to other groups with multivoltine or more complex lifecycles, particularly where these involve alternation between generations and/or plant hosts (including many aphids; Harrington et al. 2007).

### Impacts of between-provenance variation in tree vigour

Tree vigour also explained significant between-provenance variation in oak gall density. In contrast, tree shape – commonly used as an indicator of physiological stress – had little impact. As with phenology, the sign of correlations between gall density and tree vigour varied among gall generations and taxa. Significant correlations with tree vigour were positive for spring sexual generation gall types, while two of three were negative for asexual generation gall types. Support was thus evenly split between the plant vigour hypothesis (Price 1991; Cornelissen et al. 2008) and the plant stress hypothesis (White 1969, 1974, 2009). One possibility is that the apparent contrast in patterns for spring and autumn generation galls could be driven by the long-known seasonal shift in oak resource investment from growth in the spring to metabolic defence in the summer (Feeny 1970). Highly vigorous trees could then be both best for spring generation galls and worst (=best defended) for autumn generation galls. Mechanisms of oak defence against gallwasps remain poorly understood.

### Predicting the impact of climate-matched planting on associated herbivores

Climate matching necessarily requires introduction of phenotypically divergent tree populations. In Europe, climate models predict warmer summers and winters (Giorgi and Coppola 2009; Jenkins et al. 2009), and climate matching will thus always involve introducing more southerly provenances that show earlier budburst (Ducousso et al. 1996; Broadmeadow et al. 2005). Under low and high emissions scenarios for 2050, Broadmeadow et al. (2005) identified climate-matched sites for five UK locations that were 0–2° and 5–12° further south, respectively (Broadmeadow et al. 2005). Our results (Fig. 2C) suggest that while the small latitudinal shifts required under a low emission scenario would probably have little impact on oak phenology, the shifts of >10° of latitude required under a high emission scenario could lead to major phenological differences between native and climate-matched trees, and significantly dissimilar gallwasp assemblages.

The widespread existence of latitude-dependent photoperiod cues in plant phenology (Aitken et al. 2008) suggests that the phenological contrasts between climate-matched and native oak populations will be shared by other plant species. The extent to which any such differences influence associated herbivores is likely to vary according to specific plant–herbivore interactions, reflecting available herbivore phenotypic plasticity and adaptive genetic diversity. We hypothesized that impacts of phenological mismatch will be greatest for interactions in which herbivore fitness is strongly correlated with successful exploitation of a narrow phenological ‘window of opportunity’ (Durant et al. 2007; Van Asch and Visser 2007; Forkner et al. 2008; Netherer and Schopf 2010). This includes many insects that feed on newly flushed leaves (Van Asch and Visser 2007; Forkner et al. 2008). However, we found no evidence of such a window for gallwasps: while they prefer to oviposit (or survive better on) young tissues, they show no strong preference for host trees at a single phenological stage. As discussed above, impacts on herbivores with multivoltine or multihost lifecycles may be complex.

The relationships we found between host plant vigour and herbivore abundance are relevant to two stages in a climate matching strategy: the expected poor performance under current climates of introduced provenances matched to predicted future conditions, and the expected decline in the performance of native provenances as climates move away from any locally adapted range. The slope of the relationship between environmental variables and vigour for a specific provenance will dictate not only the impact on associated trophic levels, but also whether climate matching with that provenance is feasible at all. The challenge for forest managers is to identify a climate-matched provenance that is able to survive as a sapling and young tree before the conditions justifying its selection have been reached and become established before native provenances fail. Climate matching is a recently developed strategy and its success at maintaining forest productivity is untested (Broadmeadow et al. 2005). Current guidelines in UK forestry advocate mixed planting of both matched and local provenances, which increases local resource heterogeneity for herbivores (Hubert and Cottrell 2007), and creates conditions that accelerate both environmental selection on host trees (Lefevre et al. 2014) and host plant-mediated selection on associated organisms.

Assisted migration is increasingly seen as a viable strategy for commercial and conservation settings (Broadmeadow et al. 2005; Stone 2010; Dawson et al. 2011). Issues cited as slowing down the implementation of climate matching as an adaptive forestry strategy include lack of operating procedures, uncertainties over which climate models to use in matching algorithms and risks associated with moving plants outside their current ranges (Williams and Dumroese 2013). Theoretical studies nevertheless suggest that such adaptive-, rather than resilience-targeted strategies may be the best way to safeguard forest systems (Buma and Wessman 2013; Lefevre et al. 2014). We would add that the community impacts of assisted migration need to be carefully considered, particularly so for foundation species such as forest trees. This requires better understanding of the relative contributions of host plant genotype and other ecological factors to the tree phenotypes exposed to herbivores (Tack et al. 2010). Geographical patterns of local adaptation and tree phenotypic variation have only been mapped in a tiny minority of species (Savolainen et al. 2007; Robakowski et al. 2012; Sork et al. 2013), and studies that target multiple provenance trial locations are required to separate genetic and environmental contributions to provenance phenotypes (Tack et al. 2010). While our study was based on a single trial, the oak phenotypic traits we analysed have been shown to be highly heritable in the sampled provenances (Sinclair 2012), so we expect the provenance effects we found to contain a significant genetic component. Given strong environmental effects on plant traits including phenology, repetition of the work in an array of trials spanning different environments is clearly desirable. Understanding herbivore numerical and adaptive responses then in turn requires understanding the impacts of both plant phenotypes and other ecological processes, including the mortality inflicted on herbivores by their natural enemies (Bidart-Bouzat and Imeh-Nathaniel 2008; Thomson et al. 2010; Van Asch et al. 2012; Rasmann et al. 2014). Much more needs to be done before forest managers can take informed decisions concerning likely impacts of climate matching on forest-associated biodiversity.

## Data archiving statement

Data for this study are available for download from the NERC Environmental Information Data Centre: http://doi.org/10/647.

## Supporting Information

**Data S1.** Petite Charnie provenance trials.

**Data S2.** Investigating the issue of non-independence of provenances.

**Data S3.** Calculating marginal *R*^2^ of a MCMCglmm model.

**Data S4.** Consistency of spring bud-burst phenology between years.

**Data S5.** An exemplar .csv data frame for use with Data S3.

## References

[b1] Aitken SN, Yeaman S, Holliday JA, Wang T, Curtis-Mclane S (2008). Adaptation, migration or extirpation: climate change outcomes for tree populations. Evolutionary Applications.

[b2] Alberto FJ, Bouffier L, Louvet J-M, Lamy J-B, Delzon S, Kremer A (2011). Adaptive responses for seed and leaf phenology in natural populations of sessile oak along an altitudinal gradient. Journal of Evolutionary Biology.

[b3] Alberto FJ, Aitken SN, Alia R, González-Martínez SC, Hänninen H, Kremer A, Lefèvre F (2013a). Potential for evolutionary responses to climate change evidence from tree populations. Global Change Biology.

[b4] Alberto FJ, Derory J, Boury C, Frigerio JM, Zimmermann NE, Kremer A (2013b). Imprints of natural selection along environmental gradients in phenology-related genes of *Quercus petraea*. Genetics.

[b5] Askew RR (1962). The distribution of galls of *Neuroterus* (Hym: Cynipidae) on oak. The Journal of Animal Ecology.

[b6] Askew RR, Anathakrishnan TN (1984). The biology of gallwasps. The Biology of Galling Insects.

[b7] Bangert RK, Whitham TG (2007). Genetic assembly rules and community phenotypes. Evolutionary Ecology.

[b8] Benjamini Y, Hochberg Y (1995). Controlling the false discovery rate: a practical and powerful approach to multiple testing. Journal of the Royal Statistical Society Series B.

[b9] Benjamini Y, Yekutieli D (2001). The control of the false discovery rate in multiple testing under dependency. Annals of Statistics.

[b10] Bernhardsson C, Robinson KM, Abreu IN, Jansson S, Albrectsen BR, Ingvarsson PK (2013). Geographic structure in metabolome and herbivore community co-occurs with genetic structure in plant defence genes. Ecology Letters.

[b11] Bidart-Bouzat MG, Imeh-Nathaniel A (2008). Global change effects on plant chemical defenses against insect herbivores. Journal of Integrative Plant Biology.

[b12] Bolte A, Ammer C, Löf M, Madsend P, Nabuurse G-J, Schallb P, Spathelff P (2009). Adaptive forest management in central Europe: climate change impacts, strategies and integrative concept. Scandinavian Journal of Forest Research.

[b13] Bower AD, Aitken SN (2008). Ecological genetics and seed transfer guidelines for *Pinus albicaulis* (Pinaceae). American Journal of Botany.

[b14] Broadmeadow MSJ, Ray D, Samuel CJA (2005). Climate change and the future for broadleaved tree species in Britain. Forestry.

[b15] Buma B, Wessman CA (2013). Forest resilience, climate change, and opportunities for adaptation: a specific case of a general problem. Forest Ecology and Management.

[b16] Cavers S, Cottrell JE (2014). The basis of resilience in forest tree species and its use in adaptive forest management in Britain. Forestry.

[b17] Cornelissen T, Fernandes GW, Vasconcellos-Neto J (2008). Size does matter: variation in herbivory between and within plants and the plant vigor hypothesis. Oikos.

[b18] Crawley MJ, Akhteruzzaman M (1988). Individual variation in the phenology of oak trees and its consequences for herbivorous insects. Functional Ecology.

[b19] Csóka G, Stone GN, Withers TM, Melika G, Raman A, Schaefer CW (2005). Biology, ecology and evolution of gall-inducing cynipidae. Biology, Ecology and Evolution of Gall-Inducing Arthropods.

[b20] Dawson TP, Jackson ST, House JI, Prentice IC, Mace GM (2011). Beyond predictions: biodiversity conservation in a changing climate. Science.

[b21] Ducousso A, Guyon JP, Kremer A (1996). Latitudinal and altitudinal variation of budburst in western populations of sessile oak (*Quercus petraea* (Matt) Liebl). Annales Des Sciences Forestières.

[b22] Dungey HS, Potts BM, Whitham TG, Li HF (2000). Plant genetics affects arthropod community richness and composition: evidence from a synthetic eucalypt hybrid population. Evolution.

[b23] Durant JM, Hjermann DO, Ottersen G, Stenseth NC (2007). Climate and the match or mismatch between predator requirements and resource availability. Climate Research.

[b24] Egan SP, Ott JR (2007). Host plant quality and local adaptation determine the distribution of a gall-forming herbivore. Ecology.

[b25] Feeny P (1970). Seasonal changes in oak leaf tannins and nutrients as a cause of spring feeding by winter moth caterpillars. Ecology.

[b200] Felsenstein J, Veuille M, Slatkin M (2002). Contrasts for a within-species comparative method. Modern Developments in Theoretical Population Genetics.

[b26] Forkner RE, Marquis RJ, Lill JT, Le Corff J (2008). Timing is everything? Phenological synchrony and population variability in leaf-chewing herbivores of *Quercus*. Ecological Entomology.

[b27] Frelich LE, Peterson RO, Dovciak M, Reich PB, Vucetich JA, Eisenhauer N (2012). Trophic cascades, invasive species and body-size hierarchies interactively modulate climate change responses of ecotonal temperate-boreal forest. Philosophical Transactions of the Royal Society of London. Series B, Biological sciences.

[b28] Gelman A (2006). Prior distributions for variance parameters in hierarchical models (comment on an Article by Browne and Draper). Bayesian Analysis.

[b29] Giorgi F, Coppola E (2009). Projections of twenty-first century climate over Europe. Erca: From the Human Dimensions of Global Environmental Change to the Observation of the Earth from Space.

[b30] Goudet J (1995). FSTAT (Version 1.2): a computer program to calculate F-statistics. Journal of Heredity.

[b31] Guichoux E, Lagache L, Wagner S, Leger P, Petit RJ (2011). Two highly validated multiplexes (12-plex and 8-plex) for species delimitation and parentage analysis in oaks (*Quercus* spp.). Molecular Ecology Resources.

[b32] Hadfield JD (2010). MCMC methods for multi-response generalized linear mixed models: the MCMCglmm R package. Journal of Statistical Software.

[b33] Hadfield JD, Nakagawa S (2010). General quantitative genetic methods for comparative biology: phylogenies, taxonomies and multi-trait models for continuous and categorical characters. Journal of Evolutionary Biology.

[b34] Hanks LM, Denno RF (1994). Local adaptation in the armored scale insect *Pseudaulacaspis-pentagona* (Homoptera, Diaspididae). Ecology.

[b35] Harper LJ, Schönrogge K, Lim KY, Francis P, Lichtenstein CP (2004). Cynipid galls: insect-induced modifications of plant development create novel plant organs. Plant Cell and Environment.

[b36] Harrington R, Clark SJ, Welham SJ, Verrier PJ, Denholm CH, Hulle M, Maurice D (2007). Environmental change and the phenology of European aphids. Global Change Biology.

[b37] Hearn J, Stone GN, Bunnefeld L, Nicholls JA, Barton NH, Lohse K (2014). Likelihood-based inference of population history from low-coverage de novo genome assemblies. Molecular Ecology.

[b38] Heiberger RM (2013). HH: Statistical Analysis and Data Display: Heiberger and Holland.

[b39] Hersch-Green EI, Turley NE, Johnson MT (2011). Community genetics: what have we accomplished and where should we be going?. Philosophical Transactions of the Royal Society of London. Series B, Biological sciences.

[b40] Hoegh-Guldberg O, Hughes L, Mcintyre S, Lindenmayer DB, Parmesan C, Possingham HP, Thomas CD (2008). Assisted colonization and rapid climate change. Science.

[b41] Hubert J, Cottrell J (2007). The Role of Forest Genetic Resources in Helping British Forests to Respond to Climate Change.

[b42] IPCC (2013). Climate Change 2013. The Physical Science Basis. Working Group I Contribution to the Fifth Assessment Report of the Intergovernmental Panel on Climate Change.

[b43] Jenkins GJ, Murphy JM, Sexton DMH, Lowe JA, Jones P, Kilsby GC (2009). UK Climate Projections: Briefing Report.

[b44] Kennedy CEJ, Southwood TRE (1984). The number of species of insects associated with British trees – a re-analysis. Journal of Animal Ecology.

[b45] Kerstes NAG, De Jong PW (2011). Detection of refuge from enemies through phenological mismatching in multitrophic interactions requires season-wide estimation of host abundance. Evolutionary Ecology.

[b46] Laube J, Sparks TH, Estrella N, Hofler J, Ankerst DP, Menzel A (2014). Chilling outweighs photoperiod in preventing precocious spring development. Global Change Biology.

[b47] Lefevre F, Boivin T, Bontemps A, Courbet F, Davi H, Durand-Gillmann M, Fady B (2014). Considering evolutionary processes in adaptive forestry. Annals of Forest Science.

[b48] Lindenmayer DB, Banks SC, Laurance WF, Franklin JF, Likens GE (2014). Broad decline of populations of large old trees. Conservation Letters.

[b49] Lindner M, Maroschek M, Netherer S, Kremer A, Barbati A, Garcia-Gonzalo J, Seidl R (2010). Climate change impacts, adaptive capacity, and vulnerability of European forest ecosystems. Forest Ecology and Management.

[b50] Lunt ID, Byrne M, Hellmann JJ, Mitchell NJ, Garnette ST, Hayward M W, Marting TG (2013). Using assisted colonisation to conserve biodiversity and restore ecosystem function under climate change. Biological Conservation.

[b201] Matheron G (1963). Principles of geostatistics. Economic Geology.

[b51] McEwan RW, Dyer JM, Pederson N (2011). Multiple interacting ecosystem drivers: toward an encompassing hypothesis of oak forest dynamics across eastern North America. Ecography.

[b52] Mopper S (2005). Phenology – how time creates spatial structure in endophagous insect populations. Annales Zoologici Fennici.

[b53] Mopper S, Stiling P, Landau K, Simberloff D, Van Zandt P (2000). Spatiotemporal variation in leafminer population structure and adaptation to individual oak trees. Ecology.

[b54] Nakagawa S (2004). A farewell to Bonferroni: the problems of low statistical power and publication bias. Behavioral Ecology.

[b55] Nakagawa S, Schielzeth H (2013). A general and simple method for obtaining R^2^ from generalized linear mixed-effects models. Methods in Ecology and Evolution.

[b56] Netherer S, Schopf A (2010). Potential effects of climate change on insect herbivores in European forests-general aspects and the pine processionary moth as specific example. Forest Ecology and Management.

[b57] Ohlberger J, Thackeray SJ, Winfield IJ, Maberly SC, Vollestad LA (2014). When phenology matters: age-size truncation alters population response to trophic mismatch. Proceedings of the Royal Society B-Biological Sciences.

[b58] Parmesan C, Yohe G (2003). A globally coherent fingerprint of climate change impacts across natural systems. Nature.

[b59] Pearse IS, Karban R (2013). Leaf drop affects herbivory in oaks. Oecologia.

[b60] Petanidou T, Kallimanis AS, Sgardelis SP, Mazaris AD, Pantis JD, Waser NM (2014). Variable flowering phenology and pollinator use in a community suggest future phenological mismatch. Acta Oecologica.

[b61] Petit RJ, Csaikl UM, Bordacs S, Burg K, Coart E, Cottrell J, van Dam B (2002). Chloroplast DNA variation in European white oaks – phylogeography and patterns of diversity based on data from over 2600 populations. Forest Ecology and Management.

[b62] Price PW (1991). The plant vigor hypothesis and herbivore attack. Oikos.

[b63] Rasmann S, Pellissier L, Defossez E, Jactel H, Kunstler G (2014). Climate-driven change in plant–insect interactions along elevation gradients. Functional Ecology.

[b64] Redfern M, Shirley P (2002). British Plant Galls: Identification of Galls on Plants and Fungi.

[b65] Robakowski P, Li Y, Reich PB (2012). Local ecotypic and species range-related adaptation influence photosynthetic temperature optima in deciduous broadleaved trees. Plant Ecology.

[b66] Savolainen O, Pyhäjärvi T, Knürr T (2007). Gene flow and local adaptation in trees. Annual Review of Ecology Evolution and Systematics.

[b67] Savolainen O, Lascoux M, Merila J (2013). Ecological genomics of local adaptation. Nature Reviews Genetics.

[b68] Schönrogge K, Harper LJ, Lichtenstein CP (2000). The protein content of tissues in cynipid galls (Hymenoptera: Cynipidae): similarities between cynipid galls and seeds. Plant Cell and Environment.

[b900] Sinclair FH (2012). Community Level Consequences of Adaptive Management Through Climate Matching: Oak Galls as a Model System.

[b69] Singer MC, Parmesan C (2010). Phenological asynchrony between herbivorous insects and their hosts: signal of climate change or pre-existing adaptive strategy?. Philosophical Transactions of the Royal Society of London B: Biological Sciences.

[b70] Sork VL, Stowe KA, Hochwender C (1993). Evidence for local adaptation in closely adjacent subpopulations of Northern Red Oak (*Quercus rubra* L) expressed as resistance to leaf herbivores. The American Naturalist.

[b71] Sork VL, Aitken SN, Dyer RJ, Eckert AJ, Legendre P, Neale DB (2013). Putting the landscape into the genomics of trees: approaches for understanding local adaptation and population responses to changing climate. Tree Genetics and Genomes.

[b72] Spittlehouse DL, Stewart RB (2003). Adaptation to climate change in forest management. Journal of Ecosystems and Management.

[b73] Stone R (2010). Home, home outside the range?. Science.

[b74] Stone GN, Schönrogge K (2003). The adaptive significance of insect gall morphology. Trends in Ecology and Evolution.

[b75] Stone GN, Schönrogge K, Atkinson RJ, Bellido D, Pujade-Villar J (2002). The population biology of oak gallwasps (Hymenoptera: Cynipidae). Annual Review of Entomology.

[b76] Stone GN, Nee S, Felsenstein J (2011). Controlling for non-independence in comparative analysis of patterns across populations within species. Philosophical Transactions of the Royal Society of London B: Biological Sciences.

[b77] Tack AJM, Roslin T (2010). Overrun by the neighbors: landscape context affects strength and sign of local adaptation. Ecology.

[b78] Tack AJ, Ovaskainen O, Pulkkinen P, Roslin T (2010). Spatial location dominates over host plant genotype in structuring an herbivore community. Ecology.

[b79] Thomson LJ, Macfadyen S, Hoffmann AA (2010). Predicting the effects of climate change on natural enemies of agricultural pests. Biological Control.

[b80] Vakkari P, Blom A, Rusanen M, Raisio J, Toivonen H (2006). Genetic variability of fragmented stands of pedunculate oak (*Quercus robur*) in Finland. Genetica.

[b81] Van Asch M, Visser ME (2007). Phenology of forest caterpillars and their host trees: the importance of synchrony. Annual Review of Entomology.

[b82] Van Asch M, Salis L, Holleman LJM, Van Lith B, Visser ME (2012). Evolutionary response of the egg hatching date of a herbivorous insect under climate change. Nature Climate Change.

[b83] Vatka E, Rytkonen S, Orell M (2014). Does the temporal mismatch hypothesis match in boreal populations?. Oecologia.

[b84] Visser ME (2008). Keeping up with a warming world; assessing the rate of adaptation to climate change. Proceedings of the Royal Society of London B: Biological Sciences.

[b85] Walther GR, Post E, Convey P, Menzel A, Parmesan C, Beebee TJC, Fromentin J-M (2002). Ecological responses to recent climate change. Nature.

[b86] Weir BS, Cockerham CC (1984). Estimating F-statistics for the analysis of population-structure. Evolution.

[b87] White TCR (1969). An index to measure weather-induced stress of trees associated with outbreaks of psyllids in Australia. Ecology.

[b88] White TCR (1974). Hypothesis to Explain outbreaks of looper caterpillars, with special reference to populations of *Selidosema**suavis* in a Plantation of *Pinus-radiata* in New-Zealand. Oecologia.

[b89] White TCR (2009). Plant vigour versus plant stress: a false dichotomy. Oikos.

[b90] Whitham TG, Gehring CA, Lamit LJ, Wojtowicz T, Evans LM, Keith AR, Smith DS (2012). Community specificity: life and afterlife effects of genes. Trends in Plant Science.

[b91] Williams MI, Dumroese RK (2013). Preparing for climate change: forestry and assisted migration. Journal of Forestry.

[b92] Wimp GM, Martinsen GD, Floate KD, Bangert RK, Whitham TG (2005). Plant genetic determinants of arthropod community structure and diversity. Evolution.

[b93] Yukawa J (2000). Synchronization of gallers with host plant phenology. Population Ecology.

