# Supplementary material for: Impacts of local adaptation of forest trees on associations with herbivorous insects: implications for adaptive forest management
Source: Evol Appl. 2015 Oct 13;8(10):972–87. doi: 10.1111/eva.12329 (PMC4662346; doi:10.1111/eva.12329)
Supplement: Supplementary file 1 [file eva0008-0972-sd1.docx]

**Data S1**

**Petite Charnie provenance trials**

*The provenance trial environment*

The La Petite Charnie provenance trial was established by the French government’s National Institute for Agricultural Research (INRA). It contains almost 200,000 individual trees, representing 103 provenances of *Q. petraea* and 9 provenances of *Q. robur* from across much of their natural range. La Petite Charnie has a mean elevation of 140m, and its climate is typically Atlantic, temperate and wet. The geological substratum is composed principally of red sandstone, schist and lens of clay (Bacilieri & Krémer, 1995). The trial is surrounded by mature oak forest including both *Q. petraea* and *Q. robur*, mixed with beech (*Fagus sylvatica*), ash (*Fraxinus excelcior*) and hornbeam (*Carpinus betulus*). Trees native to La Petite Charnie are not represented in the trial, the closest represented population being Forêt de Bercé, 50km away.

*Establishment and design of the INRA provenance trial*

To capture natural diversity present in each contributing provenance, at each source site INRA collected acorns from at least 50 points separated by 30m in 1986, 1987, 1989, and 1992. The acorns were grown in the public nursery at Guemene-Penfao in Brittany, northwest France. At three years of age, trees of a single cohort were planted into a ‘tranche’ site that had been cleared and tilled during the previous year. The La Petite Charnie trial contains four tranches each containing a cohort of trees of the same age, numbered 1, 2, 4, & 5, planted in the early months of 1990, 1991, 1993, and 1995 respectively. Each tranche contains a unique combination of provenances and is further subdivided into several soil zones of approximately equal size, based on the soil description and associated plant communities prior to planting (Ducousso, Guyon, & Krémer, 1996).

Within each soil zone, provenances are represented by two or three distinct plots (parcelles), each a replicate set of 24 trees planted in four columns of six trees, with spacing of 1.75 meters between trees within columns and three meters between columns. Plots of 8 different randomly selected provenances are aggregated into blocks, with the position of blocks randomised within soil zones, to allow efficient statistical separation of plot, block, soil-zone, and provenance effects. As we focused on a subset of provenances, the effect of block was not considered, and the position of plots within soil zones was treated as if random.

*Selection of study provenances and trees*

We selected 20 provenances whose origins maximise available diversity in source climate and geographic distribution (see Table S1, below). To avoid comparing trees of different ages, this study used provenances represented in tranche 4, planted in 1993, which contains the greatest geographical range of provenances. In the first year of our sampling (2008), these trees were 15 years old, and up to 5m in height. For each provenance, we sampled trees from 10 plots, giving 200 sampled plots. From each plot, we surveyed 12 of the 24 trees present for cynipid galls, giving a total across all 20 provenances of 2400 sampled trees. To minimise edge effects, trees were preferentially selected from the two internal columns within each parcelle, but the nearest alternative tree was used if an internal tree had died. All 2400 trees were surveyed in each of our 4 surveys, except for the 40 parcelles in soil zone 5, which could not be sampled in Autumn 2008 due to time constraints. Because of the distribution of provenances across soil types, this had no impact on the representation of provenances in our analysis.

The tree phenotypic traits diameter at breast height (*DBH*), spring budburst (*Budburst*), and tree form (*Form*) were recorded for all trees at the La Petite Charnie trials by INRA researchers. Budburst phenology was scored during a single survey in 1995. Diameter at breast height and Form were recorded during winter 2001. The criteria for scoring spring budburst and tree form are illustrated in Figure S1.

(a)


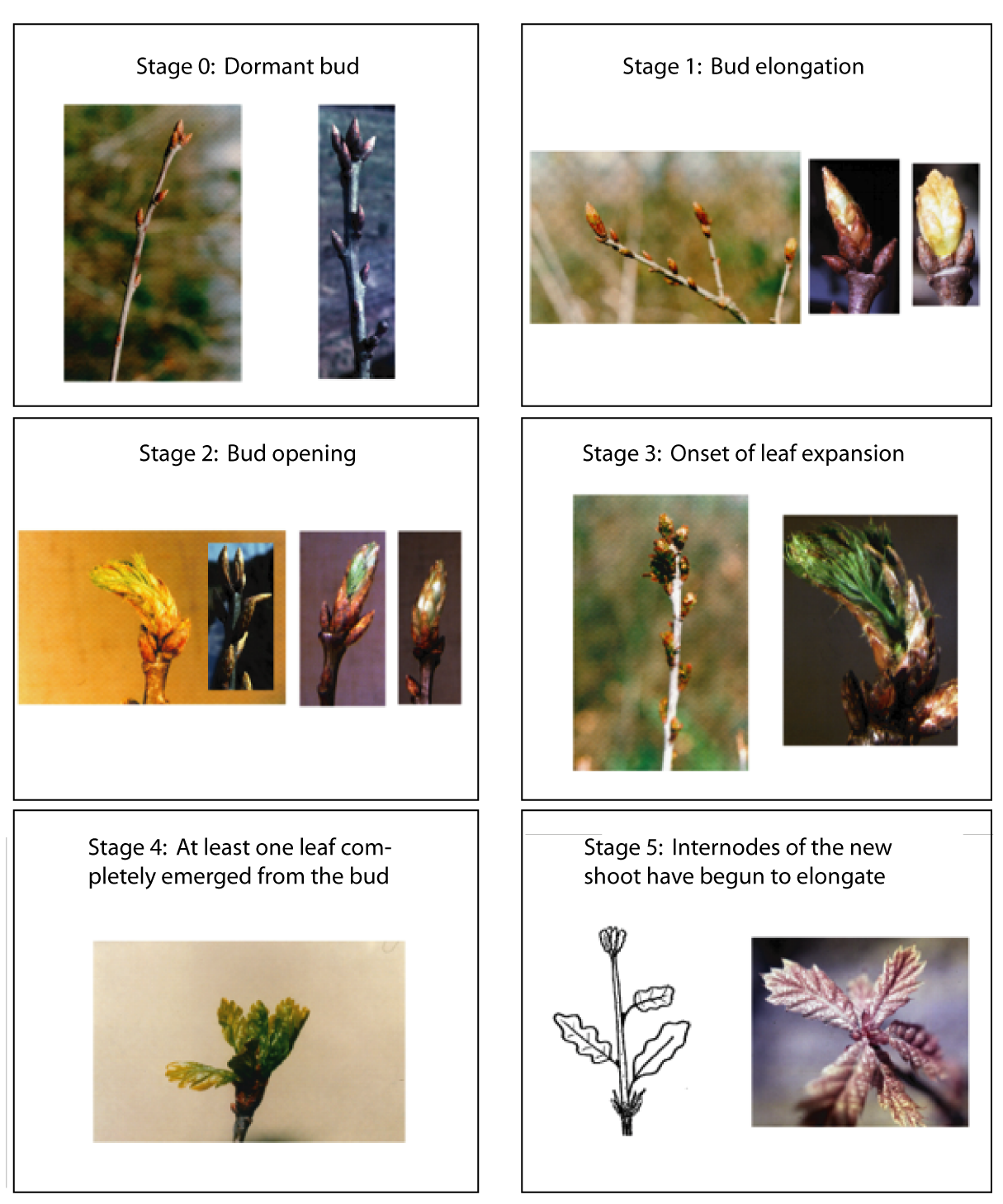


(b)


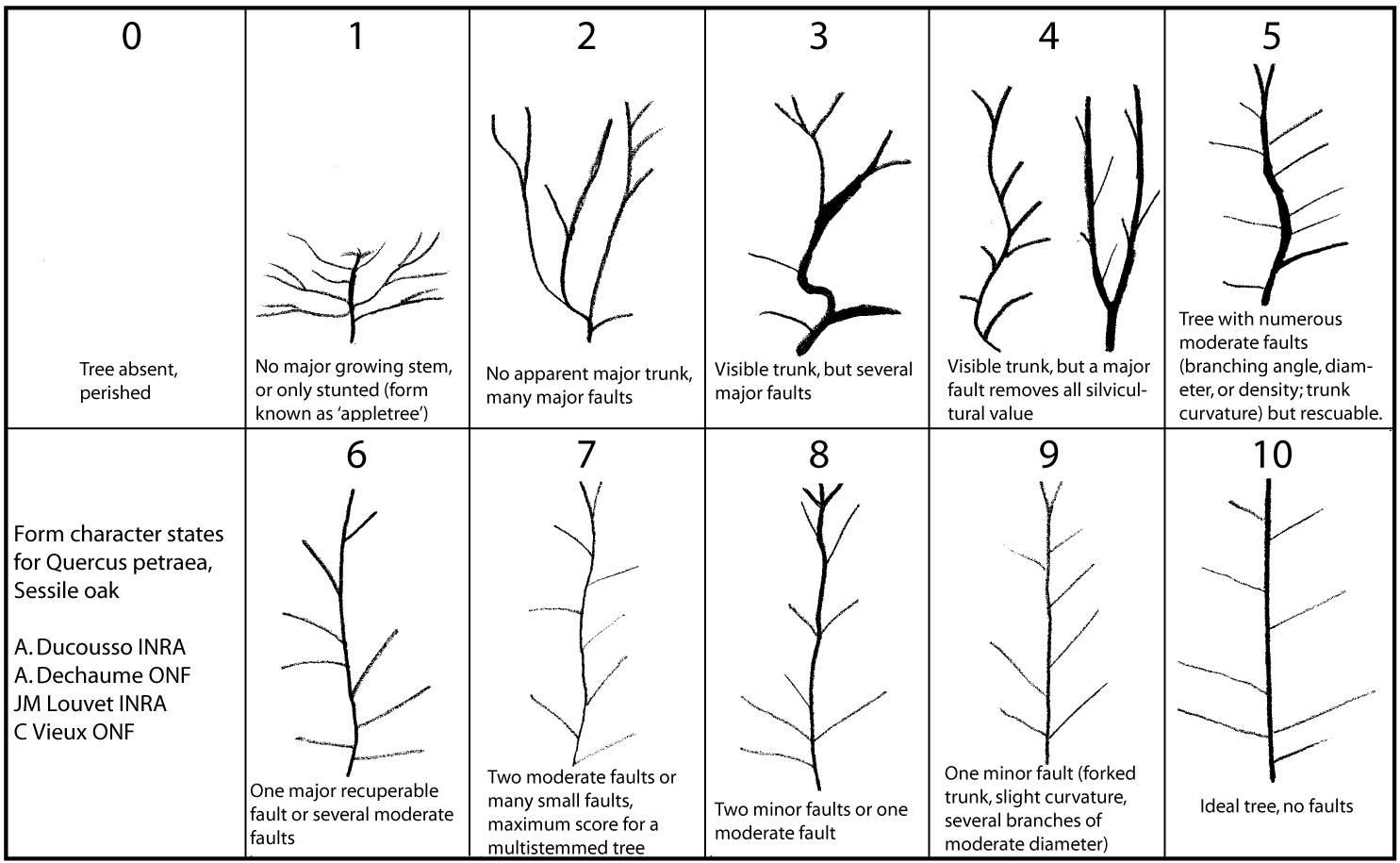


**Figure S1.** Illustration of the scored phenotypic states used by INRA to record: (a) spring budburst phenology; and (b) tree form, as a measure of health.

**Table S1.**  Summary of the source climates and geographic origins of the 20 selected provenances. For geographic distribution, see main text Fig. 1. Precipitation and temperature data were obtained from the WorldClim database (Hijmans et al. 2005), which interpolates to 1 km resolution based on weather station records from 1950-2000. Data were available for each month of the year based on averages across all available years. In the climate matching study of Broadmeadow et al (2005), sites were matched in terms of monthly precipitation, mean temperature, and diurnal temperature range, with the recent data for planting sites adjusted to incorporate predicted changes in summer (May-October) and winter (November-April) climate. In order to reflect the methods of Broadmeadow et al. (2005), monthly climate data for the study provenances were either summed (for precipitation) or averaged (for temperatures) to give the following summary variables:

· SummerPrec – The total precipitation (in mm) falling between May and October.

· WinterPrec – The total precipitation (in mm) falling between November and April.

· SummerTemp – Mean temperature (in C °) between May and October

· WinterTemp – Mean temperature (in C °) between November and April

| Code | Forest | Country | Longitude | Latitude | SummerPrec (mm) | WinterPrec (mm) | SummerTemp (°C) | WinterTemp (°C) |
| --- | --- | --- | --- | --- | --- | --- | --- | --- |
| 179 | Sycow | Poland | 17.93 | 51.18 | 372 | 202 | 14.54 | 1.87 |
| 181 | Horbylunde | Denmark | 9.41 | 56.13 | 419 | 355 | 12.64 | 1.81 |
| 184 | Telavi | Georgia | 45.47 | 41.88 | 553 | 287 | 14.61 | 1.18 |
| 185 | Blakeney | UK | -2.50 | 51.78 | 378 | 398 | 13.90 | 5.74 |
| 194 | Soudrain | France | 2.38 | 46.95 | 377 | 348 | 16.20 | 6.08 |
| 201 | La Haie Renaut | France | 4.95 | 48.67 | 369 | 310 | 15.23 | 4.79 |
| 210 | Saint Germain | France | 2.08 | 48.90 | 335 | 315 | 15.30 | 5.57 |
| 211 | Prémery | France | 3.60 | 47.20 | 402 | 350 | 15.73 | 5.51 |
| 217 | Bercé | France | 0.39 | 47.81 | 331 | 378 | 15.53 | 5.89 |
| 225 | Still | France | 7.25 | 48.58 | 460 | 386 | 13.79 | 2.72 |
| 230 | Romersberg | France | 6.73 | 48.82 | 392 | 343 | 15.13 | 4.14 |
| 233 | Vachères | France | 5.63 | 43.98 | 377 | 402 | 16.08 | 5.46 |
| 237 | Réno Valdieu | France | 0.67 | 48.50 | 332 | 354 | 14.86 | 5.51 |
| 245 | Etangs | France | 4.96 | 46.93 | 415 | 353 | 16.63 | 5.48 |
| 248 | Klostermarienberg | Austria | 16.57 | 47.41 | 424 | 219 | 15.73 | 2.85 |
| 249 | Bolu | Turkey | 31.67 | 40.92 | 281 | 470 | 13.78 | 2.13 |
| 250 | Cochem | Germany | 7.05 | 50.08 | 373 | 322 | 14.22 | 3.58 |
| 252 | Johanneskreuz | Germany | 7.83 | 49.40 | 414 | 363 | 13.48 | 2.50 |
| 255 | Spakensehl | Germany | 10.60 | 52.80 | 367 | 290 | 14.03 | 2.78 |
| 257 | Wolfgang | Germany | 9.05 | 50.15 | 360 | 281 | 15.61 | 4.17 |

References

Bacilieri, R., & Krémer, A. (1995). Genetic, morphological, ecological and phenological differentiation between Quercus petraea (Matt.) Liebl. and Quercus robur L. in a mixed stand of northwest of France. *Silvae Genetica*, *44*(1), 1–10.

Broadmeadow M.S.J., Ray D. & Samuel C.J.A. (2005). Climate change and the future for broadleaved tree species in Britain. *Forestry*, *78*, 145-161.

Ducousso, A., Guyon, J. P., & Krémer, A. (1996). Latitudinal and altitudinal variation of bud burst in western populations of sessile oak (*Quercus petraea* (Matt) Liebl ). *Annals of Forest Science*, *53*, 775–782.

Hijmans, R. J., S. E. Cameron, J. L. Parra, P. G. Jones, and A. Jarvis. 2005. Very high resolution interpolated climate surfaces for global land areas. *International Journal of Climatology, 25*, 1965-1978.
