# Supplementary material for: Impacts of local adaptation of forest trees on associations with herbivorous insects: implications for adaptive forest management
Source: Evol Appl. 2015 Oct 13;8(10):972–87. doi: 10.1111/eva.12329 (PMC4662346; doi:10.1111/eva.12329)
Supplement: Supplementary file 2 [file eva0008-0972-sd2.doc]

**Data S2**

**Investigating the issue of non-independence of provenances**

*Summary*

A common shortcoming of comparative studies of intraspecies populations is that the populations are treated as statistically independent entities. This is unlikely to be true, as populations that are genetically similar through gene-flow or common ancestry can be expected to co-vary in traits independently of any population-specific effects (such as impacts of local selection) (Felsenstein 2002; Stone et al. 2011), and failure to address this statistically could lead to erroneous inferences. In our study we investigated the robustness of our inferences by incorporating into our models various scenarios of provenance covariance based on *F_ST_*  – a widely used measure of genetic differentiation between populations. Under the assumptions of an infinite islands population genetic model, and for diallelic markers, *F_ST_* varies between 0 (no differentiation) and 1 (complete differentiation). Our premise is that we expect populations showing lowest differentiation to show highest variance covariance. Implications of using alternative models are considered below.

The results of these investigations suggest that models that treat provenances as statistically independent entities (i) provide a useful minimum estimate for the influence of tree provenance on gall abundance; and (ii) infer relationships between tree phenotypic traits and gall abundance that are robust to issues of non-independence of provenances. Here we provide further information on the methods, rationale, and results of our investigations of this issue.

*Estimating provenance differentiation*

Genotypic data were available for 17 of the 20 study provenances in the form of genotypes for individual trees across 10 co-dominant diploid microsatellite loci, determined by multiplex PCR reactions, with alleles defined by their fragment lengths. Samples ranged in size from 21 to 29 trees per provenance. The microsatellite loci had been screened for null alleles through progeny testing and the dataset exhibited no sign of linkage disequilibrium or deviation from Hardy-Weinberg equilibrium (*p* < 0.05 after Bonferroni adjustment). A summary of the diversity and differentiation at each locus is provided in Table S2.1.

**Table S2.1.** Estimates of population genetic parameters for 10 microsatellite loci, including: observed (*H_o_*) and expected (*H_e_*) levels of heterozygosity, averaged across populations and calculated using GenAlEx version 6.3; Wrights F-statistics (*F_it_, F_st_, & F_is_*) and *R_st_*, estimated for each locus and summarised across loci following Weir and Cockerham (1984) using FSTAT version 2.9.3.2; and *D_est_*, Jost’s measure of genetic differentiation between populations (Jost 2008), estimated for each locus and summarised as the approximated harmonic mean across loci using the program SMOGD version 1.2.5 (Crawford 2010). Confidence intervals for each locus were based on 1000 bootstrap replicates, with re-sampling of individuals. Confidence intervals for global estimates were based on 1000 bootstrap replicates with re-sampling of loci.

| **Locus** | **No. of alleles** | **Mean allelic richness** | ***H_o_*** | ***H_e_*** | ***F_IT_*** | ***F_ST_*** | ***F_IS_*** | ***R_ST_*** | **Min *D_EST_* 95% CI** | ***D_EST_*** | **Max *D_EST_* 95% CI** |
| --- | --- | --- | --- | --- | --- | --- | --- | --- | --- | --- | --- |
| PIE239 | 16 | 4.93 | 0.282 | 0.272 | 0.031 | 0.045 | -0.014 | 0.022 | 0.014 | 0.018 | 0.04 |
| PIE102 | 15 | 8.75 | 0.766 | 0.733 | -0.009 | 0.016 | -0.025 | -0.004 | 0.069 | 0.049 | 0.135 |
| PIE242 | 12 | 8.31 | 0.818 | 0.804 | 0.019 | 0.015 | 0.004 | 0.016 | 0.11 | 0.075 | 0.201 |
| PIE243 | 13 | 6.35 | 0.712 | 0.728 | 0.049 | 0.014 | 0.035 | 0.022 | 0.06 | 0.04 | 0.135 |
| PIE227 | 8 | 4.29 | 0.607 | 0.605 | 0.045 | 0.028 | 0.017 | 0.018 | 0.053 | 0.049 | 0.109 |
| PIE223 | 11 | 5.92 | 0.596 | 0.577 | 0.015 | 0.026 | -0.011 | 0.029 | 0.042 | 0.038 | 0.09 |
| PIE258 | 28 | 15.31 | 0.897 | 0.884 | 0.023 | 0.018 | 0.005 | 0.009 | 0.258 | 0.175 | 0.358 |
| PIE152 | 23 | 10.8 | 0.830 | 0.805 | 0.004 | 0.015 | -0.011 | 0.035 | 0.119 | 0.076 | 0.195 |
| PIE267 | 12 | 8.09 | 0.785 | 0.772 | 0.027 | 0.023 | 0.005 | -0.007 | 0.114 | 0.088 | 0.196 |
| PIE020 | 11 | 6.36 | 0.667 | 0.659 | 0.028 | 0.026 | 0.002 | 0.037 | 0.062 | 0.059 | 0.14 |
|  |  |  |  |  |  |  |  |  |  |  |  |
| Across loci | - | - | 0.696 | 0.684 | 0.022 | 0.021 | 0.002 | 0.0138 | - | 0.047 | - |
| 95% CI | - | - | - | - | ± 0.010 | ± 0.04 | ± 0.010 | - | - | - | - |

For the subset of 17 provenances, a pair-wise matrix of *F_ST_* across all loci was estimated using FSTAT version 2.8.3.2 (Goudet 1995). Any negative values were adjusted to zero. Pair-wise matrices of geographic distance (in km) were obtained using GenAlEx version 6.3 (Peakall and Smouse 2006) for both the subset of 17 provenances and the full set of 20 provenances. There was a significant correlation between the *F_ST_* and geographic distance matrices for the set of 17 provenances – as revealed by Mantel testing in the *adegenet* R package (*p* = 0.01, number of repeats = 999) – indicating a pattern of isolation by distance. The relationship between *F_ST_* and geographic distance (GD) was parameterised using linear regression (Fig. S2.1) as:

*Equation 1. F_ST_ =* 0.001155 + (0.00001881 × GD)

This function was applied to the geographic distance matrices for the sets of 17 and 20 provenances to obtain estimates of a pseudo-*F_ST_* (the estimates derived in this way will henceforth be referred to as *GeoF_ST_*). Matrices for modelling of provenance covariance were estimated as either 1-*F_ST_* or 1-*GeoF_ST_,* such that expected covariance was greatest between provenances with lowest genetic differentiation, and populations separated by a distance of zero are have an expected covariance of 1.

*Impacts of using genetic measures of differentiation other than F_ST_*

Other measures of genetic differentiation exist that are related to, or estimate, *F_ST_* (such as Jost’s *D_EST_* and Slatkin’s *R_ST_* - see Meirmans and Hedrick 2011 for a recent review). One feature of *F_ST_* is that for highly variable markers such as microsatellites the maximum value may be somewhat less than 1, such that our use of (1- *F_ST_*) may overestimate expected population covariance. Further, metrics such as Slatkins R*_ST_* that incorporate a mutation model for microsatellites also tend to result in higher estimates of differentiation than simple *F_ST_* (Neigel 2001), such that use of 1-R*_ST_* would result in lower estimates of population covariance.

To explore the consequences of using alternative measures, we estimated *F_ST_*, *D_EST_* and *R_ST_* for each locus and across all loci (see Table S2.1). As expected, all *D_EST_* values were >*F_ST_*, while the relationship between *R_ST_* and *F_ST_* varied among loci with *R_ST_* showing a slightly lower mean across loci. Use of *R_ST_* or *F_ST_* will this result in higher estimates of covariance than *D_EST_*.

We generated pairwise estimates of *D_EST_* and R_ST_ between the set of 17 provenances and examined their relationships with pairwise matrices of *F_ST_* and geographic distance (pairwise matrices were estimated from the microsatellite data using the program SMOGD version 1.2.5 (Crawford 2010) for *D_EST_*, and Arlequin version 3.5.1.3 (Excoffier *et al*, 2005) for *R_ST_*; negative values were addjusted to zero). The three measures of differentiation were all strongly correlated with one another (and particularly *F_ST_* with *D_EST_*) and with geographic distance (see Table S2.2). We chose to use *F_ST_* because (i) all values in the pairwise matrix are both similar to *R_ST_* and positive (in contrast to *R_ST_*) and (ii) the generally low *F_ST_* values mean that this measure should provide an upper bound estimate of provenance covariance. We infer that results supported using each of 1-*F_ST_* and identity covariance matrices will be robust to use of such alternative covariance measures.

**Table S2.2.** Summary of relationships between pairwise matrices of geographic distance, and the genetic differentiation measures *D_EST_, R_ST_* & F_ST_ for set of 17 provenances. Table cells show values for the measure in the left-most column modelled against the matrix listed in the top row. For each correlation we show the *p*-value of correlation between matrices estimated from Mantel testing in the *Adegenet* R package (with 999 repeats), and the R^2^ and slope parameter from linear regressions between matrices.

|  | Geo-distance | | *D_EST_* | | *R_ST_* | |
| --- | --- | --- | --- | --- | --- | --- |
|  | *p* | R^2^ | *p* | R^2^ | *p* | R^2^ |
| Geo-distance | NA | NA |  |  |  |  |
| *D_EST_* | 0.024 | 0.602 | NA | NA |  |  |
| *R_ST_* | 0.003 | 0.4796 | 0.001 | 0.647 | NA | NA |
| *F_ST_* | 0.018 | 0.5596 | 0.001 | 0.9365 | 0.001 | 0.6717 |


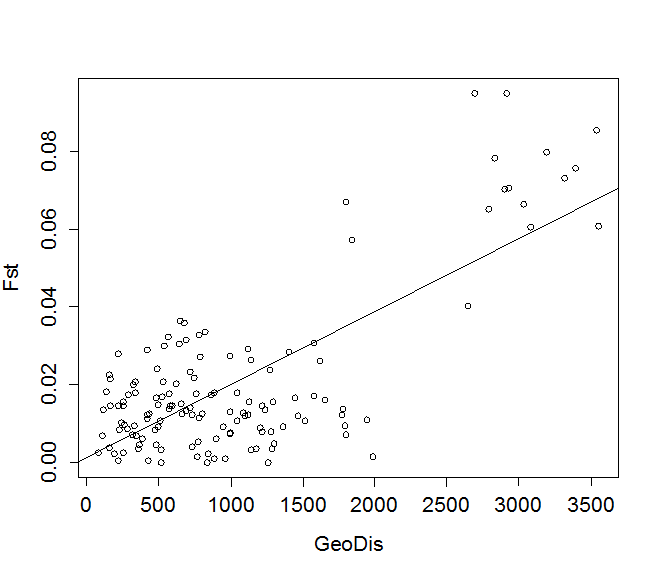


**Figure S2.1**. (a) The relationship between pairwise *F_ST_* and geographic separation for the 17 provenance dataset. The fitted line is a linear regression (see Equation 1 for parameters).

**Table S2.3.** Pairwise matrices of: (a) 1- *F_ST_* for 17 provenance data set; (b) geographic distance (in km) for the 20 provenance dataset; & (c) 1-*GeoF_ST_* for 20 provenance dataset. All matrices are symmetric about the diagonal but for clarity only the lower halves are shown here. Provenance (Prov) codes follow the original INRA codes used in Table 1 of the main article.

(a)

| **Prov Code** | **179** | **181** | **194** | **201** | **210** | **211** | **217** | **225** | **233** | **237** | **245** | **248** | **249** | **250** | **252** | **255** | **257** |
| --- | --- | --- | --- | --- | --- | --- | --- | --- | --- | --- | --- | --- | --- | --- | --- | --- | --- |
| **179** | 1 |  |  |  |  |  |  |  |  |  |  |  |  |  |  |  |  |
| **181** | 0.987 | 1 |  |  |  |  |  |  |  |  |  |  |  |  |  |  |  |
| **194** | 0.991 | 0.992 | 1 |  |  |  |  |  |  |  |  |  |  |  |  |  |  |
| **201** | 0.988 | 0.999 | 0.993 | 1 |  |  |  |  |  |  |  |  |  |  |  |  |  |
| **210** | 0.986 | 0.974 | 0.972 | 0.980 | 1 |  |  |  |  |  |  |  |  |  |  |  |  |
| **211** | 0.984 | 0.997 | 0.982 | 1.000 | 0.985 | 1 |  |  |  |  |  |  |  |  |  |  |  |
| **217** | 0.999 | 0.991 | 0.990 | 0.997 | 0.992 | 0.995 | 1 |  |  |  |  |  |  |  |  |  |  |
| **225** | 0.992 | 0.983 | 0.982 | 0.985 | 0.986 | 0.988 | 0.998 | 1 |  |  |  |  |  |  |  |  |  |
| **233** | 0.969 | 0.972 | 0.976 | 0.983 | 0.964 | 0.971 | 0.977 | 0.970 | 1 |  |  |  |  |  |  |  |  |
| **237** | 0.989 | 0.996 | 0.998 | 0.992 | 0.985 | 0.997 | 0.998 | 0.996 | 0.978 | 1 |  |  |  |  |  |  |  |
| **245** | 0.989 | 0.984 | 0.983 | 0.998 | 0.994 | 0.996 | 1.000 | 0.993 | 0.979 | 0.989 | 1 |  |  |  |  |  |  |
| **248** | 1.000 | 0.987 | 0.983 | 0.995 | 0.974 | 0.983 | 0.993 | 0.989 | 0.976 | 0.988 | 0.985 | 1 |  |  |  |  |  |
| **249** | 0.943 | 0.930 | 0.927 | 0.940 | 0.924 | 0.920 | 0.939 | 0.922 | 0.905 | 0.915 | 0.934 | 0.933 | 1 |  |  |  |  |
| **250** | 0.986 | 0.986 | 0.980 | 0.991 | 0.968 | 0.985 | 0.988 | 0.979 | 0.968 | 0.988 | 0.988 | 0.988 | 0.930 | 1 |  |  |  |
| **252** | 0.997 | 0.982 | 0.988 | 0.991 | 0.969 | 0.979 | 0.998 | 0.993 | 0.964 | 0.988 | 0.989 | 0.992 | 0.935 | 0.987 | 1 |  |  |
| **255** | 1.000 | 0.994 | 0.988 | 0.995 | 0.982 | 0.993 | 1.000 | 0.986 | 0.971 | 0.991 | 0.994 | 0.999 | 0.960 | 0.991 | 0.995 | 1 |  |
| **257** | 0.973 | 0.985 | 0.967 | 0.983 | 0.973 | 0.987 | 0.987 | 0.990 | 0.967 | 0.991 | 0.986 | 0.982 | 0.905 | 0.986 | 0.978 | 0.982 | 1 |

**(b)**

| **Prov Code** | **179** | **181** | **184** | **185** | **194** | **201** | **210** | **211** | **217** | **225** | **230** | **233** | **237** | **245** | **248** | **249** | **250** | **252** | **255** | **257** |
| --- | --- | --- | --- | --- | --- | --- | --- | --- | --- | --- | --- | --- | --- | --- | --- | --- | --- | --- | --- | --- |
| **179** | 0 |  |  |  |  |  |  |  |  |  |  |  |  |  |  |  |  |  |  |  |
| **181** | 1088 | 0 |  |  |  |  |  |  |  |  |  |  |  |  |  |  |  |  |  |  |
| **184** | 3181 | 4235 | 0 |  |  |  |  |  |  |  |  |  |  |  |  |  |  |  |  |  |
| **185** | 2273 | 1409 | 5423 | 0 |  |  |  |  |  |  |  |  |  |  |  |  |  |  |  |  |
| **194** | 1790 | 1281 | 4817 | 763 | 0 |  |  |  |  |  |  |  |  |  |  |  |  |  |  |  |
| **201** | 1469 | 961 | 4553 | 898 | 344 | 0 |  |  |  |  |  |  |  |  |  |  |  |  |  |  |
| **210** | 1780 | 1142 | 4873 | 602 | 219 | 320 | 0 |  |  |  |  |  |  |  |  |  |  |  |  |  |
| **211** | 1651 | 1179 | 4684 | 848 | 138 | 222 | 253 | 0 |  |  |  |  |  |  |  |  |  |  |  |  |
| **217** | 1985 | 1362 | 5046 | 546 | 241 | 516 | 224 | 363 | 0 |  |  |  |  |  |  |  |  |  |  |  |
| **225** | 1221 | 865 | 4299 | 1141 | 571 | 256 | 576 | 434 | 768 | 0 |  |  |  |  |  |  |  |  |  |  |
| **230** | 1271 | 858 | 4359 | 1078 | 526 | 199 | 517 | 392 | 714 | 64 | 0 |  |  |  |  |  |  |  |  |  |
| **233** | 1575 | 1404 | 4435 | 1252 | 489 | 525 | 674 | 422 | 721 | 539 | 549 | 0 |  |  |  |  |  |  |  |  |
| **237** | 1941 | 1287 | 5024 | 507 | 257 | 476 | 163 | 356 | 83 | 732 | 675 | 746 | 0 |  |  |  |  |  |  |  |
| **245** | 1514 | 1129 | 4531 | 989 | 287 | 193 | 388 | 154 | 517 | 313 | 287 | 335 | 508 | 0 |  |  |  |  |  |  |
| **248** | 428 | 1235 | 3255 | 2174 | 1579 | 1299 | 1619 | 1442 | 1800 | 1044 | 1105 | 1273 | 1772 | 1292 | 0 |  |  |  |  |  |
| **249** | 1843 | 2931 | 1537 | 3969 | 3318 | 3079 | 3395 | 3190 | 3553 | 2829 | 2892 | 2913 | 3538 | 3035 | 1802 | 0 |  |  |  |  |
| **250** | 1216 | 716 | 4345 | 1079 | 624 | 281 | 568 | 499 | 782 | 167 | 144 | 692 | 731 | 419 | 1098 | 2898 | 0 |  |  |  |
| **252** | 1139 | 760 | 4247 | 1179 | 664 | 330 | 642 | 530 | 846 | 111 | 138 | 647 | 802 | 420 | 996 | 2793 | 115 | 0 |  |  |
| **255** | 834 | 388 | 4014 | 1461 | 1119 | 776 | 1041 | 994 | 1262 | 595 | 614 | 1117 | 1202 | 901 | 883 | 2642 | 495 | 484 | 0 |  |
| **257** | 994 | 658 | 4126 | 1297 | 822 | 484 | 787 | 688 | 997 | 264 | 297 | 779 | 950 | 577 | 887 | 2690 | 223 | 159 | 338 | 0 |

**(c)**

| **Prov**  **Code** | **179** | **181** | **184** | **185** | **194** | **201** | **210** | **211** | **217** | **225** | **230** | **233** | **237** | **245** | **248** | **249** | **250** | **252** | **255** | **257** |
| --- | --- | --- | --- | --- | --- | --- | --- | --- | --- | --- | --- | --- | --- | --- | --- | --- | --- | --- | --- | --- |
| **179** | 1 |  |  |  |  |  |  |  |  |  |  |  |  |  |  |  |  |  |  |  |
| **181** | 0.978 | 1 |  |  |  |  |  |  |  |  |  |  |  |  |  |  |  |  |  |  |
| **184** | 0.939 | 0.919 | 1 |  |  |  |  |  |  |  |  |  |  |  |  |  |  |  |  |  |
| **185** | 0.956 | 0.972 | 0.897 | 1 |  |  |  |  |  |  |  |  |  |  |  |  |  |  |  |  |
| **194** | 0.965 | 0.975 | 0.908 | 0.984 | 1 |  |  |  |  |  |  |  |  |  |  |  |  |  |  |  |
| **201** | 0.971 | 0.981 | 0.913 | 0.982 | 0.992 | 1 |  |  |  |  |  |  |  |  |  |  |  |  |  |  |
| **210** | 0.965 | 0.977 | 0.907 | 0.988 | 0.995 | 0.993 | 1 |  |  |  |  |  |  |  |  |  |  |  |  |  |
| **211** | 0.968 | 0.977 | 0.911 | 0.983 | 0.996 | 0.995 | 0.994 | 1 |  |  |  |  |  |  |  |  |  |  |  |  |
| **217** | 0.962 | 0.973 | 0.904 | 0.989 | 0.994 | 0.989 | 0.995 | 0.992 | 1 |  |  |  |  |  |  |  |  |  |  |  |
| **225** | 0.976 | 0.983 | 0.918 | 0.977 | 0.988 | 0.994 | 0.988 | 0.991 | 0.984 | 1 |  |  |  |  |  |  |  |  |  |  |
| **230** | 0.975 | 0.983 | 0.917 | 0.979 | 0.989 | 0.995 | 0.989 | 0.991 | 0.985 | 0.998 | 1 |  |  |  |  |  |  |  |  |  |
| **233** | 0.969 | 0.972 | 0.915 | 0.975 | 0.990 | 0.989 | 0.986 | 0.991 | 0.985 | 0.989 | 0.989 | 1 |  |  |  |  |  |  |  |  |
| **237** | 0.962 | 0.975 | 0.904 | 0.989 | 0.994 | 0.990 | 0.996 | 0.992 | 0.997 | 0.985 | 0.986 | 0.985 | 1 |  |  |  |  |  |  |  |
| **245** | 0.970 | 0.978 | 0.914 | 0.980 | 0.993 | 0.995 | 0.992 | 0.996 | 0.989 | 0.993 | 0.993 | 0.993 | 0.989 | 1 |  |  |  |  |  |  |
| **248** | 0.991 | 0.976 | 0.938 | 0.958 | 0.969 | 0.974 | 0.968 | 0.972 | 0.965 | 0.979 | 0.978 | 0.975 | 0.966 | 0.975 | 1 |  |  |  |  |  |
| **249** | 0.964 | 0.944 | 0.970 | 0.924 | 0.936 | 0.941 | 0.935 | 0.939 | 0.932 | 0.946 | 0.944 | 0.944 | 0.932 | 0.942 | 0.965 | 1 |  |  |  |  |
| **250** | 0.976 | 0.985 | 0.917 | 0.979 | 0.987 | 0.994 | 0.988 | 0.989 | 0.984 | 0.996 | 0.996 | 0.986 | 0.985 | 0.991 | 0.978 | 0.944 | 1 |  |  |  |
| **252** | 0.977 | 0.985 | 0.919 | 0.977 | 0.986 | 0.993 | 0.987 | 0.989 | 0.983 | 0.997 | 0.996 | 0.987 | 0.984 | 0.991 | 0.980 | 0.946 | 0.997 | 1 |  |  |
| **255** | 0.983 | 0.992 | 0.923 | 0.971 | 0.978 | 0.984 | 0.979 | 0.980 | 0.975 | 0.988 | 0.987 | 0.978 | 0.976 | 0.982 | 0.982 | 0.949 | 0.990 | 0.990 | 1 |  |
| **257** | 0.980 | 0.986 | 0.921 | 0.974 | 0.983 | 0.990 | 0.984 | 0.986 | 0.980 | 0.994 | 0.993 | 0.984 | 0.981 | 0.988 | 0.982 | 0.948 | 0.995 | 0.996 | 0.992 | 1 |

*Specifying provenance covariance in MCMCglmm*

In the MCMCglmm R package (Hadfield 2010), the variance co-variance matrix of a random effect in a generalised linear mixed model (GLMM) is by default an identity matrix – i.e. a square matrix with ones on the main diagonal and zeros everywhere else. An alternative variance co-variance matrix can be specified using the following code:

Ainv <- as(solve(*M*), "dgCMatrix")

model.1 <- MCMCglmm(y ~ 1, random = Provenance, ginverse=list(Provenance=Ainv))

where ‘Provenance’ is a factor with a unique level for each provenance, and ‘*M’* is a square positive definite matrix of the estimated covariance between all pairs of provenances. This will not work if *M* is not positive definite (i.e. if one or more of the eigen values of the matrix are negative), but in such cases the columns of the matrix that have negative eigen values can be discarded, and the un-normalised eigen vectors of the remaining columns (V_i_) used to specify variance co-variance of the random effect as:

model.2 <- MCMCglmm(y ~ 1, random = ~idv(V_1_+V_2_+V_3_…))

The 1- *F_ST_* matrix for our 17 provenance dataset was not positive definite, and so the model.2 approach above was applied wherever covariance was estimated as 1- *F_ST_*. The *GeoF_ST_* matrix was positive definite for both the 17 and the 20 provenance datasets, and so the model.1 approach was applied wherever covariance was estimated as 1-*GeoF_ST_*.

*Investigating the influence of tree provenance on gall abundance*

The size of the influence of tree provenance on gall abundance was estimated as the variance component of the provenance random effect(s) from a GLMM (see section ‘Methods: Statistical analysis’ in the main article). This was initially investigated for 14 gall types for five dataset/covariance matrix combinations as follows:

(i) The subset of 17 provenances for which genotypic data were available, using an identity matrix as the provenance covariance matrix. This identity model assumes provenances to be statistically entirely independent;

(ii) The same 17 provenances, using a covariance matrix estimated as 1-*F_ST_*;

(iii) The same 17 provenances, using a covariance matrix estimated as 1-*GeoF_ST_*;

(iv) The full set of 20 provenances using an identity matrix; and

(v) The full set of 20 provenances where covariance is estimated as 1-*GeoF_ST_*.

In the modelling of combinations ii–v, provenance featured in two random effects – one with a default identity co-variance matrix and the other with the specified co-variance matrix – and the overall effect of provenance was estimated from the sum of the variances of these two random effects (see section ‘Methods: Statistical analysis’ in the main paper for rationale).

There was very little difference in results between the 1-*F_ST_* and 1-*GeoF_ST_* approaches for the 17 provenance dataset, supporting the viability of *GeoF_ST_* as a proxy for *F_ST_.* There was also little difference between the identity approaches for the 17 and 20 provenance datasets, although the 1-*GeoF_ST_* approach for the 20 provenance dataset generally resulted in higher estimations of the provenance effect than the equivalent approach for the 17 provenance dataset. For all gall-types, using a 1-*F_ST_* or *1-GeoF_ST_* co-variance matrix substantially increased the proportion of variation attributed to the effect of provenance (Figure S2.2).

If 1-*F_ST_* accurately describes provenance co-variance then these results imply that the influence of tree provenance on gall abundance is substantially greater than would be inferred from models that treat provenances as statistically independent entities. However, although *F_ST_* is a widely used measure of genetic differentiation between populations and we consider it to be a sensible tool for investigating the non-independence of provenances, the question of how well it describes true patterns of co-variation due to migration and shared ancestry is undetermined and beyond the scope of this study. In particular, while 1-*F_ST_* is likely to describe the pattern of covariance, with more genetically differentiated populations having lower covariance, it may not describe the magnitude of the covariance. Estimates of *F_ST_* between pairings of our study provenances did not exceed 0.1, and hence the degree of provenance co-variance specified by the 1-*F_ST_* or 1-*GeoF_ST_* matrices are high – typically greater than 0.9 and often approaching 1 (Figure S2.2a & c). Such a scenario where even the most geographically remote provenances co-vary by 90% or more is a stark contrast to the identity matrix scenario where all provenance pairings, including some less than 100 km apart, are treated as independent. Our intention here was not to accurately identify and model population non-independence, but rather to check that the inferences drawn from our results are not erroneous through failure to control for non-independence. We therefore considered it prudent to investigate further, using variations on the 1-*GeoF_ST_* covariance matrix.

As we observed evidence of isolation by distance in our study system, an alternative intermediate scenario could be that provenance covariance decreases with increasing geographic distance between provenances, becoming essentially independent above a certain distance threshold. As previously described, the relationship between *F_ST_* and geographic distance was parameterised (see equation 1) and used to estimate *GeoF_ST_* from geographic distance. To create covariance matrices where *GeoF_ST_* increased linearly and reached 1 (i.e. independence) at a specified geographic distance, we used Equation 1 to develop the following function:

*Equation 2. GeoF_ST_ =* 0.001155 + (((1-0.001155)/ID) × GD)

Where ID is the independence distance – i.e. the distance (in km) at which provenances become independent from one another. This was applied to the geographic distance matrix for our 20 provenance data set (Table S2.3b) with ID values of 500, 1000, 2000, & 4000. For use as provenance co-variance matrices the main diagonal was changed to contain all zeros, and values of greater than 1 were adjusted to 1 before a 1-*GeoF_ST_* transformation was applied

The variance components of the provenance random effects were estimated for the 14 gall types with each of the 4 independence distance based co-variance matrices (Figure S2.3). In all cases the estimated effect of provenance was intermediate between the identity model approach and the unadjusted *GeoF_ST_* approach. For each gall type the provenance effect increased with increasing independence distance, which makes intuitive sense: where independence distance is lowest, we expect the pattern among a widely distributed set of samples (as we have) to most closely match that obtained assuming that samples are wholly independent.

**Figure S2.2.** The proportion of variation attributed to the effect of provenance for each of 14 gall-types for different variance-covariance models, indicated by symbol colour: the 17 provenance dataset where co-variance is defined by an identity matrix (blue circles), a 1-*Fst* matrix (red circles), or a 1-*GeoFst* matrix (yellow circles); and for the 20 provenance dataset where co-variance is defined by an identity matrix (black circles), or a 1-*GeoFst* matrix (grey circles). Vertical bars represent 95 % confidence intervals for each mean.


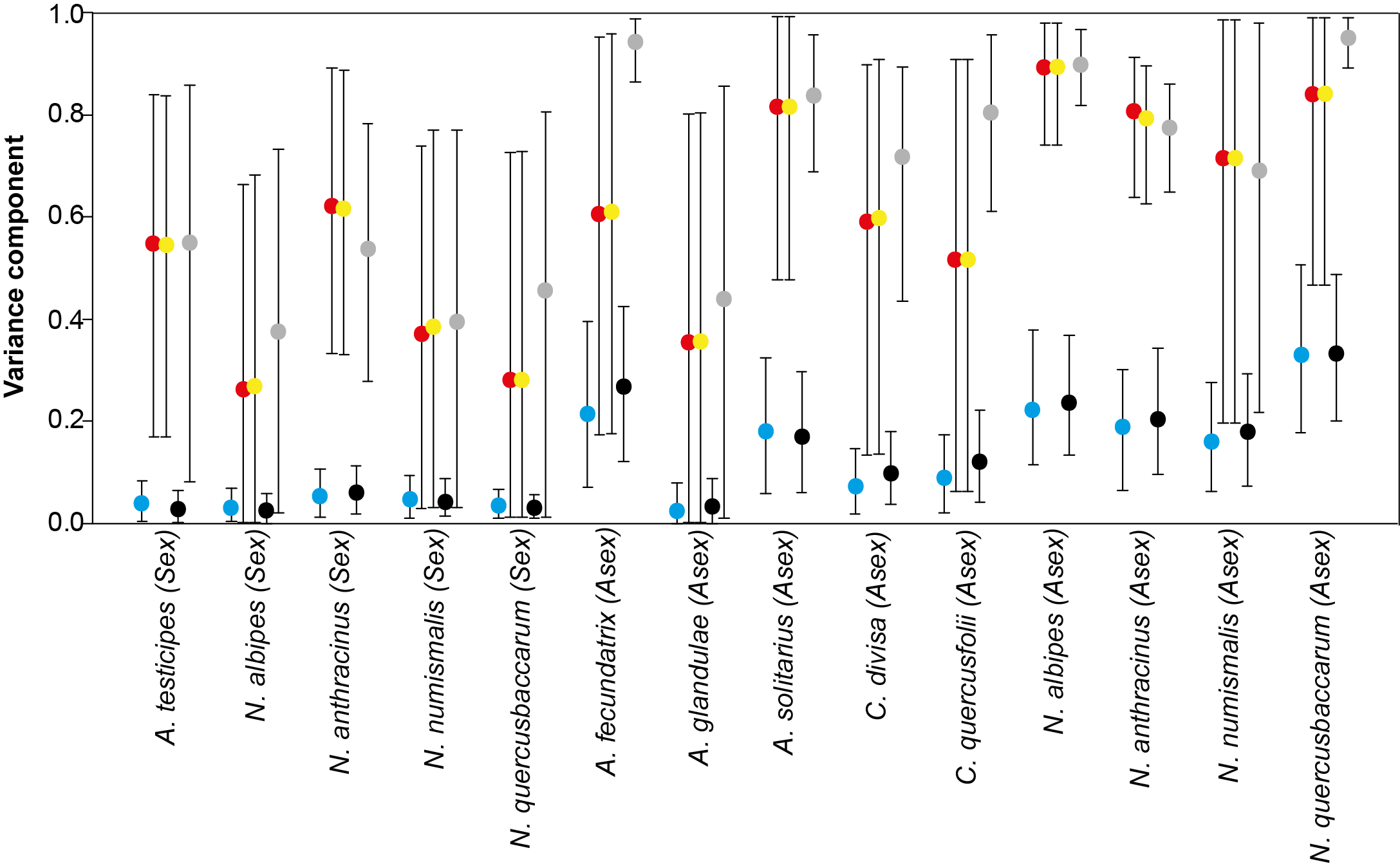


**Figure S2.3.** The proportion of variation in gall abundance attributed to the effect of provenance for the 20 provenance dataset where co-variance is defined by: an identity matrix (black circles); 1-*GeoFst* matrices with independence at distances of 500 km (red circles), 1000 km (yellow circles), 2000 km (blue circles), and 4000 km (purple circles), and an unadjusted 1-*GeoFst* matrix (grey circles). Vertical bars represent 95 % confidence intervals for each mean.


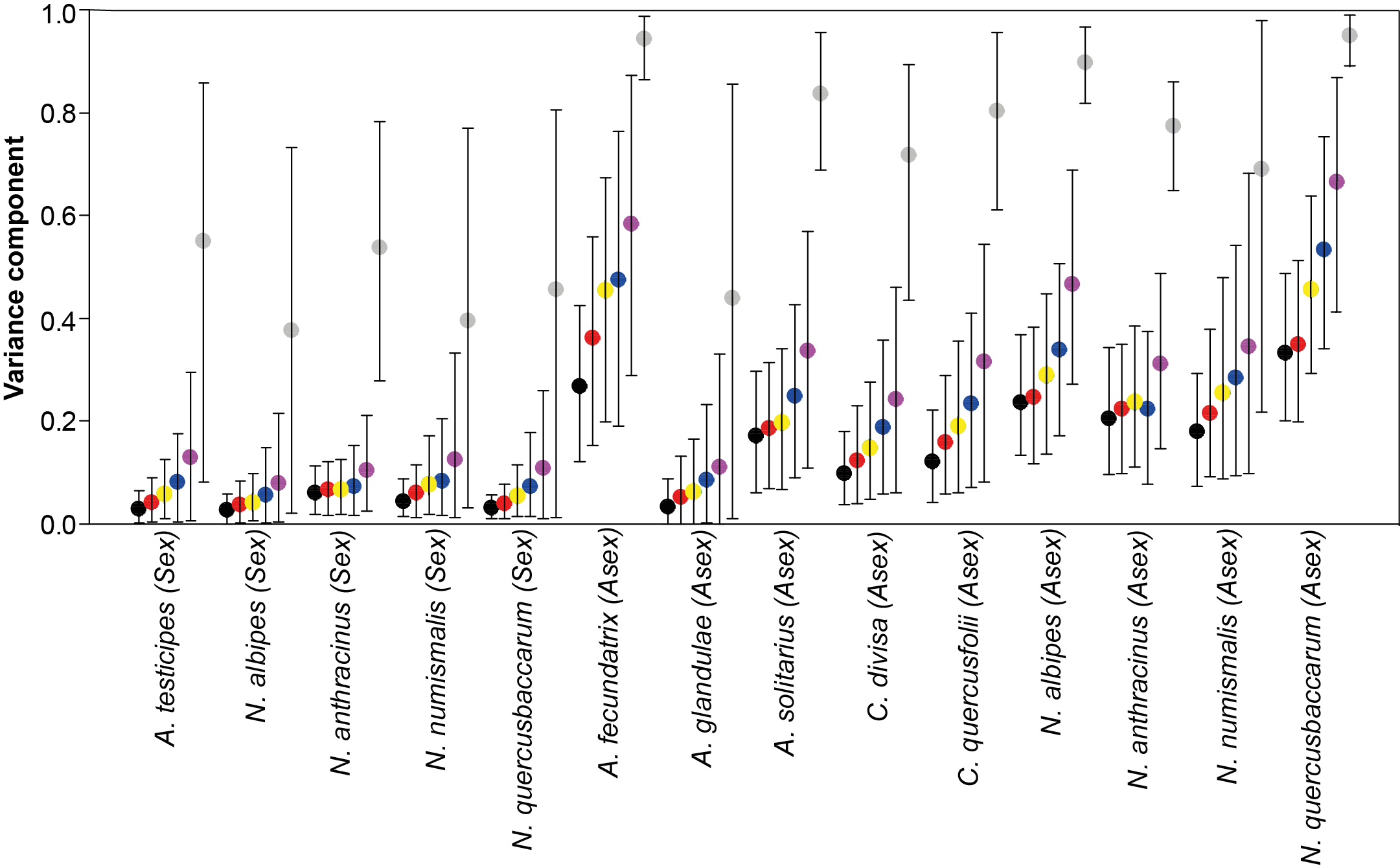


*Investigating the effect of tree phenotypic traits*

The analyses of tree phenotypic traits as predictors of variation in gall abundance (for methods see section ‘Methods: Statistical analysis’ in the main article) was conducted for 14 gall types for the five dataset/covariance matrix combinations described in the previous section (for results see Table S2.4). Parameter estimates differed little between different covariance approaches to the same dataset (i.e. datasets of 17 or 20 provenances), but more so between approaches with different datasets. For the two covariance approaches with the 20 provenance dataset, there were no instances where a predictor was significant for one but not the other (n=30, see Table S2.4), although the level of significance did differ in 5 instances. For the three covariance approaches with the 17 provenance dataset, there was one instance where a predictor was significant for the 1-*GeoF_ST_* covariance matrix but not for the 1-*F_ST_* or *Identity* covariance matrices. In most instances, a predictor that was significant for at least one dataset/covariance matrix combination was significant for all five combinations (22 / 31). The consistency of these results suggests that inferences about the effect of tree phenotypic traits on gall abundances are robust across analyses.

**Table S2.4.** Estimates of fixed effect coefficients for year and tree phenotypic traits for 14 gall types for each of 5 dataset/covariance matrix combinations. Significance was determined from the probability that the posterior distribution of the fixed effect coefficients did not include zero (pMCMC), and significant relationships are shown in bold (significance codes are: * = 95 %, ** = 99 %, *** = 99.9 %).

| **Gall Type** | **Covariance matrix : no. of provenances in dataset** | **Year** | ***Form*** | ***DBH*** | ***Budburst*** | ***Budburst^2^*** |
| --- | --- | --- | --- | --- | --- | --- |
|  |  |  |  |  |  |  |
| *A. testacipes* (Sex) | Identity : 17 | **1.121***** | 0.007 | **0.002*** | 0.019 | 0.032 |
|  | 1-*Fst* : 17 | **1.122***** | 0.009 | **0.002*** | 0.014 | 0.032 |
|  | 1-*GeoFst :* 17 | **1.120***** | 0.008 | **0.002*** | 0.021 | 0.031 |
|  | Identity : 20 | **1.048***** | 0.016 | **0.002*** | 0.013 | 0.015 |
|  | 1-*GeoFst* : 20 | **1.051***** | 0.016 | **0.002**** | 0.016 | 0.015 |
|  |  |  |  |  |  |  |
| *N. albipes* (Sex) | Identity : 17 | **1.922***** | 0.025 | 0.000 | -0.014 | -0.019 |
|  | 1-*Fst* : 17 | **1.924***** | 0.027 | 0.000 | -0.012 | -0.018 |
|  | 1-*GeoFst :* 17 | **1.923***** | 0.025 | 0.000 | -0.013 | -0.018 |
|  | Identity : 20 | **1.944***** | 0.026 | 0.000 | -0.023 | -0.011 |
|  | 1-*GeoFst* : 20 | **1.947***** | 0.026 | 0.000 | -0.018 | -0.010 |
|  |  |  |  |  |  |  |
| *N. anthracinus* (Sex) | Identity : 17 | **-0.673***** | 0.017 | 0.000 | 0.022 | 0.001 |
|  | 1-*Fst* : 17 | **-0.674***** | 0.018 | 0.000 | 0.019 | 0.001 |
|  | 1-*GeoFst :* 17 | **-0.674***** | 0.017 | 0.000 | **0.023*** | 0.001 |
|  | Identity : 20 | **-0.638***** | 0.018 | 0.000 | **0.020*** | -0.001 |
|  | 1-*GeoFst* : 20 | **-0.636***** | 0.016 | 0.000 | **0.024*** | -0.001 |
|  |  |  |  |  |  |  |
| *N. numismalis* (Sex) | Identity : 17 | **2.751***** | 0.024 | **0.001*** | **0.112***** | -0.018 |
|  | 1-*Fst* : 17 | **2.752***** | 0.025 | **0.001*** | **0.113***** | -0.018 |
|  | 1-*GeoFst :* 17 | **2.751***** | 0.025 | **0.001*** | **0.112***** | -0.018 |
|  | Identity : 20 | **2.764***** | 0.004 | **0.001***** | **0.107***** | **-0.020*** |
|  | 1-*GeoFst* : 20 | **2.763***** | 0.004 | **0.001**** | **0.104***** | **-0.020*** |
|  |  |  |  |  |  |  |
| *N. quercusbaccarum* (Sex) | Identity : 17 | **0.376***** | -0.005 | 0.000 | **0.083***** | -0.003 |
|  | 1-*Fst* : 17 | **0.375***** | -0.004 | 0.000 | **0.082***** | -0.003 |
|  | 1-*GeoFst :* 17 | **0.376***** | -0.004 | 0.000 | **0.080***** | -0.002 |
|  | Identity : 20 | **0.387***** | -0.002 | 0.000 | **0.076***** | -0.005 |
|  | 1-*GeoFst* : 20 | **0.387***** | -0.003 | 0.000 | **0.077***** | -0.005 |
|  |  |  |  |  |  |  |
| *A. fecundatrix* (Asex) | Identity : 17 | **1.109***** | -0.049 | **-0.004*** | **-0.352***** | **0.123*** |
|  | 1-*Fst* : 17 | **1.114***** | -0.054 | **-0.004*** | **-0.344***** | **0.124**** |
|  | 1-*GeoFst :* 17 | **1.115***** | -0.053 | **-0.005*** | **-0.351***** | **0.122*** |
|  | Identity : 20 | **1.115***** | -0.021 | -0.003 | **-0.330**** | **0.107*** |
|  | 1-*GeoFst* : 20 | **1.117***** | -0.025 | -0.003 | **-0.334***** | **0.109**** |
|  |  |  |  |  |  |  |
| *A. glandulae* (Asex) | Identity : 17 | **1.091***** | -0.022 | -0.002 | -0.105 | 0.037 |
|  | 1-*Fst* : 17 | **1.096***** | -0.021 | -0.002 | -0.104 | 0.036 |
|  | 1-*GeoFst :* 17 | **1.067***** | -0.025 | -0.002 | -0.100 | 0.036 |
|  | Identity : 20 | **1.129***** | 0.016 | -0.003 | **-0.140*** | 0.008 |
|  | 1-*GeoFst* : 20 | **1.144***** | 0.009 | -0.003 | **-0.132*** | 0.009 |
|  |  |  |  |  |  |  |
| *A. solitarius* (Asex) | Identity : 17 | 0.074 | -0.037 | 0.000 | -0.011 | -0.022 |
|  | 1-*Fst* : 17 | 0.080 | -0.036 | 0.000 | -0.015 | -0.020 |
|  | 1-*GeoFst :* 17 | 0.075 | -0.033 | 0.000 | -0.012 | -0.023 |
|  | Identity : 20 | 0.074 | -0.017 | 0.001 | -0.006 | -0.020 |
|  | 1-*GeoFst* : 20 | 0.078 | -0.016 | 0.001 | -0.004 | -0.020 |
|  |  |  |  |  |  |  |
| *Cynips divisa* (Asex) | Identity : 17 | **-2.688*** | 0.042 | -0.001 | -0.076 | -0.012 |
|  | 1-*Fst* : 17 | **-2.688*** | 0.049 | -0.001 | -0.077 | -0.013 |
|  | 1-*GeoFst :* 17 | **-2.696*** | 0.044 | -0.001 | -0.073 | -0.011 |
|  | Identity : 20 | **-2.708*** | 0.072 | -0.001 | **-0.112*** | 0.004 |
|  | 1-*GeoFst* : 20 | **-2.706*** | 0.069 | -0.001 | **-0.104*** | 0.007 |
|  |  |  |  |  |  |  |
| *C. quercusfolii* (Asex) | Identity : 17 | **0.395**** | 0.110 | -0.001 | -0.085 | 0.060 |
|  | 1-*Fst* : 17 | **0.394**** | 0.109 | -0.001 | -0.086 | 0.062 |
|  | 1-*GeoFst :* 17 | **0.391**** | 0.111 | -0.001 | -0.080 | 0.060 |
|  | Identity : 20 | **0.429**** | **0.129*** | 0.000 | -0.069 | 0.058 |
|  | 1-*GeoFst* : 20 | **0.432***** | **0.132*** | -0.001 | -0.050 | 0.056 |
|  |  |  |  |  |  |  |
| *N. albipes* (Asex) | Identity : 17 | **-1.019***** | -0.012 | 0.001 | -0.006 | -0.019 |
|  | 1-*Fst* : 17 | **-1.018***** | -0.010 | 0.001 | -0.008 | -0.019 |
|  | 1-*GeoFst :* 17 | **-1.018***** | -0.011 | 0.001 | -0.006 | -0.019 |
|  | Identity : 20 | **-0.961***** | -0.009 | **0.001*** | -0.006 | -0.013 |
|  | 1-*GeoFst* : 20 | **-0.961***** | -0.011 | **0.001*** | -0.007 | -0.013 |
|  |  |  |  |  |  |  |
| *N. anthracinus* (Asex) | Identity : 17 | -3.505*** | 0.000 | 0.000 | -0.009 | 0.013 |
|  | 1-*Fst* : 17 | -3.505*** | 0.001 | 0.000 | -0.011 | 0.013 |
|  | 1-*GeoFst :* 17 | -3.505*** | 0.001 | 0.000 | -0.011 | 0.013 |
|  | Identity : 20 | -3.467*** | 0.014 | 0.000 | -0.017 | 0.016 |
|  | 1-*GeoFst* : 20 | -3.466*** | 0.013 | 0.000 | -0.016 | 0.016 |
|  |  |  |  |  |  |  |
| *N. numismalis* (Asex) | Identity : 17 | **-0.602**** | -0.030 | **-0.009*** | **-1.123***** | 0.168 |
|  | 1-*Fst* : 17 | **-0.616**** | -0.027 | **-0.009*** | **-1.109***** | 0.165 |
|  | 1-*GeoFst :* 17 | **-0.616***** | -0.029 | **-0.009*** | **-1.102***** | **0.170*** |
|  | Identity : 20 | **-0.688***** | 0.035 | **-0.009*** | **-1.111***** | **0.181*** |
|  | 1-*GeoFst* : 20 | **-0.684***** | 0.024 | **-0.009*** | **-1.094***** | **0.185*** |
|  |  |  |  |  |  |  |
| *N. quercusbaccarum* (Asex) | Identity : 17 | **-0.477***** | -0.010 | **-0.002***** | **-0.169***** | 0.025 |
|  | 1-*Fst* : 17 | **-0.477***** | -0.006 | **-0.002**** | **-0.168***** | 0.025 |
|  | 1-*GeoFst :* 17 | **-0.476***** | -0.008 | **-0.002**** | **-0.169***** | 0.025 |
|  | Identity : 20 | **-0.509***** | -0.007 | **-0.002***** | **-0.175***** | **0.025*** |
|  | 1-*GeoFst* : 20 | **-0.506***** | -0.006 | **-0.002***** | **-0.174***** | **0.026*** |
|  |  |  |  |  |  |  |

**Conclusions**

In our study we: (i) use the variance component of provenance random effect(s) in GLMMs to make inferences about the influence of tree provenance in determining gall abundance; & (ii) use the coefficients of fixed effects in GLMMs to make inferences about the role of particular tree phenotypic traits in determining gall abundance. The purpose of investigating issues of non-independence was to reassure that these inferences were not erroneous through failure to statistically account for the non-independence of provenances.

Our studied tree species *Quercus petraea* spread from several refugia following the last glacial period and current patterns of nuclear genetic diversity are considered to reflect a combination of the selection pressures acting on established populations, and wind mediated pollen flow between refugial lineages where they have met in central Europe (Kremer et al. 2002). As such, we consider it likely that current populations will exhibit a degree of non-independence due to gene-flow or common ancestry, and that the pattern of non-independence will be described by a measure of genetic differentiation such as *F_ST_*. However, the magnitude of non-independence is more difficult to determine and may not be well described by *F_ST_*. The results presented here indicate that models that treat provenances as statistically independent entities consistently result in lower estimates of the influence of tree provenance relative to models where covariance is specified based on a measure of genetic differentiation (*F_ST_*). From this, although we may not be able to accurately estimate the influence of tree provenance, we conclude that models that treat provenances as statistically independent entities provide a useful minimum estimate for the influence of tree provenance on gall abundance.

With regards to (ii), the significance of fixed effect coefficients was relatively consistent across different datasets/subsets and covariance approaches. For the full dataset of 20 provenances the same coefficients were significant (differing only slightly in value and level of significance) in approaches that modelled provenances as statistically independent, or that incorporated covariance based on *Fst.* We therefore conclude that our inferences about the relationships between tree phenotypic traits and gall abundance from models that treat provenances as statistically independent entities are robust to issues of non-independence.

**References**

Crawford NG (2010) smogd: software for the measurement of genetic diversity. *Molecular Ecology Resources*, **10**, 556-557.

Excoffier L, Laval G, Schneider S (2005) Arlequin ver. 3.0: An integrated software package for population genetics data analysis. *Evolutionary Bioinformatics Online*, **1**, 47- 50.

Felsenstein J (2002) Contrasts for a within-species comparative method. In: *Modern Developments in Theoretical Population Genetics* (eds Slatkin M, Veuille M), pp. 118-129, Oxford University Press, Oxford U.K.

Goudet J (1995) FSTAT (Version 1.2): A computer program to calculate F-statistics. *Journal of Heredity,* **86**, 485-486.

Hadfield J (2010) MCMC methods for multi-response generalized linear mixed models: the MCMCglmm R package. *Journal of Statistical Software,* **33**, 1–22.

Jost L (2008) G(ST) and its relatives do not measure differentiation. *Molecular Ecology,* **17**, 4015-4026.

Kremer A, Kleinschmit J, Cottrell J, Cundall EP, Deans JD, Ducousso A, Konig AO, Lowe AJ, Munro RC, Petit RJ, Stephan BR (2002) Is there a correlation between chloroplastic and nuclear divergence, or what are the roles of history and selection on genetic diversity in European oaks? *Forest Ecology and Management*, **156**, 75–87.

Meirmans PG, Hedrick PW (2011) Assessing population structure: FST. *Molecular Ecology Resources,* **11**, 5–18.

Neigel JE (2002) Is *F_ST_* obsolete? *Conservation Genetics,* **3**, 167–173.

Peakall R, Smouse PE (2006) GENALEX 6: genetic analysis in Excel. Population genetic software for teaching and research. *Molecular Ecology Notes,* **6**, 288–295.

Stone GN, Nee S, Felsenstein J (2011) Controlling for non-independance in comparative analysis of patterns across populations within species. *Philosophical Transactions of the Royal Society B-Biological Sciences,* **366**, 1410–1424.

Weir BS, Cockerham, CC (1984) Estimating F-statistics for the analysis of population structure. *Evolution,* **38**, 1358–1370.
