# Supplementary material for: Impacts of local adaptation of forest trees on associations with herbivorous insects: implications for adaptive forest management
Source: Evol Appl. 2015 Oct 13;8(10):972–87. doi: 10.1111/eva.12329 (PMC4662346; doi:10.1111/eva.12329)
Supplement: Supplementary file 3 [file eva0008-0972-sd3.docx]

# Data S3

# Calculating marginal R2 of a MCMCglmm model

# download supplementary file GallData.csv to use the script below

#

#'Impacts of local adaptation of forest trees on associations with herbivorous # insects, and implications for adaptive forest management.'

#

# Frazer H. Sinclair^1,3^, Graham Stone^1^, James Nicholls^1^, Stephen Cavers^2^,

# Melanie Gibbs^3^, Philip Butterill^3,4^, Stefanie Wagner, Alexis Ducousso^5^, Sophie # Gerber^5^, Rémy Petit^5^, Antoine Kremer^5^, Karsten Schönrogge^3^

# 1. Institute of Evolutionary Biology, University of Edinburgh, Edinburgh EH9 # 3JT, United Kingdom.

# 2. Centre for Ecology and Hydrology, Edinburgh EH26 0QB, United Kingdom.

# 3. Centre for Ecology and Hydrology, Wallingford OX10 8BB, United Kingdom.

# 4. Department of Zoology, University of South Bohemia, České Budějovice,

# Czech Republic.

# 5. BIOGECO, National Institute for Agricultural Research (INRA), Pierotton

# 33612, France.

#

# Running title: Oak provenance affects gall abundance

# Correspondence: K Schonrogge; email: ksc@ceh.ac.uk

#

# This script demonstrates a method for calculating Nakagawa & Schielzeth’s # (2013) marginal R2 for an MCMCglmm (Hadfield 2010) model of gall abundance # within a provenance trial experiment of sessile oak *Quercus petraea*

#

# Hadfield JD (2010) MCMC Methods for Multi-Response Generalized Linear Mixed

# Models: The MCMCglmm R Package. J. Stat. Soft. 33(2):1-22.

#

# Nakagawa S & Schielzeth H (2013) A general and simple method for

# obtainingR2from generalized linear mixed-effects models. Methods in Ecology

# and Evolution. 4(2):133-142.

###############################################################################

# A. Preparation

####################################

# clear memory

rm(list=ls())

# Open MCMCglmm package

require(MCMCglmm)

# Read Data5.csv - a data frame with 1591 rows and 5 columns, with counts of

# *Neuroterus quercusbaccarum* Asexual generation galls from 10 shoots per tree

# in each of 2 years for 89 trees, belonging to 10 different provenances and

# planted across 5 different soil zones. We suggest changing the data frame

# name to GallData at this point

GallData<-read.csv("Data5.csv",header=TRUE)

# Declare SoilZone as a factor

GallData$SoilZone<-factor(GallData$SoilZone)

# Set priors for fixed and random effects - for fixed effects, prior settings

# assume a normal distribution with a mean (mu) of 0 and a variance (V) of

# 10^8. For random effect variances, priors are parameter expanded with prior

# means (alpha.mu) of 0 and prior covariances (alpha.V) of 1000, assuming an

# Inverse Wishart distribution with the scalar parameters V=1 and nu=1.

Priors<-list(R = list(V = 1, nu = 0.002), G = list(G1 = list(V=1, nu=1, alpha.mu=0, alpha.V=1000), G2 = list(V=1, nu=1, alpha.mu=0, alpha.V=1000),G3 = list(V=1, nu=1, alpha.mu=0, alpha.V=1000)))

# B. Analysis

####################################

# Run model with no fixed effects, and ProvCode, SoilZone, and TreeID as random

# effects

modNull<-MCMCglmm(NqbAsex ~ 1, random = ~Provenance+SoilZone+TreeID, nitt=50000,burnin=5000, thin=45, data = GallData,verbose = FALSE, prior = Priors,family = "poisson")

# Run model with Year as a fixed effect, and ProvCode, SoilZone, and TreeID as

# random effects

mod1<-MCMCglmm(NqbAsex ~ Year, random = ~Provenance+SoilZone+TreeID, nitt=50000,burnin=5000, thin=45, data = GallData, saveX=T, verbose = FALSE, prior = Priors,family = "poisson")

# C. Extraction of the variance components and calculation of Nakagawa's

# marginal R2

####################################

# Extract sigma of Provenance as random effect

sigPC=summary(modNull)$Gcovariances[1]

# Extract sigma of SoilZone as random effect

sigSZ=summary(modNull)$Gcovariances[2]

# Extract sigma of TreeID as random effect

sigTID=summary(modNull)$Gcovariances[3]

# Extract sigma of Unit as random effect

sigU=summary(modNull)$Rcovariances[1]

# Extract intercept

beta0=posterior.mode(modNull$Sol[,1])

# Extract sigma/variance of year as fixed effect

sigFix=var(as.vector(posterior.mode(mod1$Sol[,2])*mod1$X[,2]))

# Calculate RsqM

RsqM =(sigFix)/(sigFix+sigPC+sigSZ+sigTID+sigU+log(1/exp(beta0)+1))

RsqM
