# Supplementary material for: Impacts of local adaptation of forest trees on associations with herbivorous insects: implications for adaptive forest management
Source: Evol Appl. 2015 Oct 13;8(10):972–87. doi: 10.1111/eva.12329 (PMC4662346; doi:10.1111/eva.12329)
Supplement: Supplementary file 4 [file eva0008-0972-sd4.docx]

**Data S4**

**Consistency of spring bud-burst phenology between years**

**Note:** the analyses presented here are drawn from the Doctoral thesis of Sinclair (2012).

Our study includes investigation of the relationship between spring bud-burst phenology and the abundance of species of herbivorous gallwasps on 2400 sessile oak trees (*Quercus petraea*). The abundances of gall wasps were surveyed during both spring and autumn of 2008 and 2009, but the spring bud-burst phenology of the trees was scored some years earlier, during the spring of 1995 (see also supplementary file Data S1). Independent studies have reported that while the timing of spring bud-burst in oaks can vary considerably between years, rankings of individual trees are relatively constant (Askew 1962, Crawley and Akhteruzzaman 1988), and in our study it is assumed that the bud-burst phenology scores from 1995 provide a reliable measure of relative phenology in 2008 & 2009. To allow testing of this assumption, measurements of bud-burst phenology were repeated on April 15^th^ 2009 (referred to as *Budburst_2009_*), following the original scoring system for a subset of trees from 8 of the study provenances (all living trees within 2 parcelles of each provenance in both soil zones 1 & 2).

*Statistical analysis & results*

Modelling was conducted in R version 2.13.0 (R Development Core Team 2011) with the *lmer* function of the *lme4* package (Bates et al. 2011). Models were fitted with a Gaussian error distribution, and with maximum likelihood estimation to allow for comparison of models with differing fixed effect structures (Bolker et al. 2009). For a sample of 582 trees where measures of spring bud-burst phenology were available from both 1995 and 2009, the relationship between these measures was investigated by modelling *Budburst_2009_* with the corresponding measurements of *Budburst_1995_* included as a fixed effect, and soil zone, parcelle, and provenance fitted as random effects (for description of these see main article and supplementary file Data S1). The significance of the relationship between the two phenology scores was assessed through the estimation of the *Budburst_1995_* fixed effect slope parameter and its confidence intervals, as derived from a posterior distribution of 50,000 samples generated using Markov Chain Monte Carlo methods with the *mcmcsamp* and *pvals.fnc* functions from the *lme4* and *LanguageR* R packages respectively (Baayen 2011, Bates et al. 2011).

Results revealed significant positive co-linearity between bud-burst phenology scores from 2009 (*Budburst_2009_*) and from 1995 (*Budburst_1995_*) (Table S4.1 and Figure S4.1). This indicates a general consistency in relative phenology between 1995 and 2009, and supports the assumption that the bud-burst phenology scores from 1995 provide a reliable measure of relative phenology in 2008 & 2009.

**Table S4.1** Summary results for modelling of relationships between phenotypic traits, showing the pair-wise combinations of response and predictor variables, and estimates of the slope parameter for predictor variables with 95% confidence intervals.

| **Response** | **Predictor** | **Slope** | **lower 95% CI** | **Upper 95% CI** |
| --- | --- | --- | --- | --- |
|  |  |  |  |  |
| *Budburst_2009_* | *Budburst_1995_* | 0.1756 | 0.122 | 0.2539 |


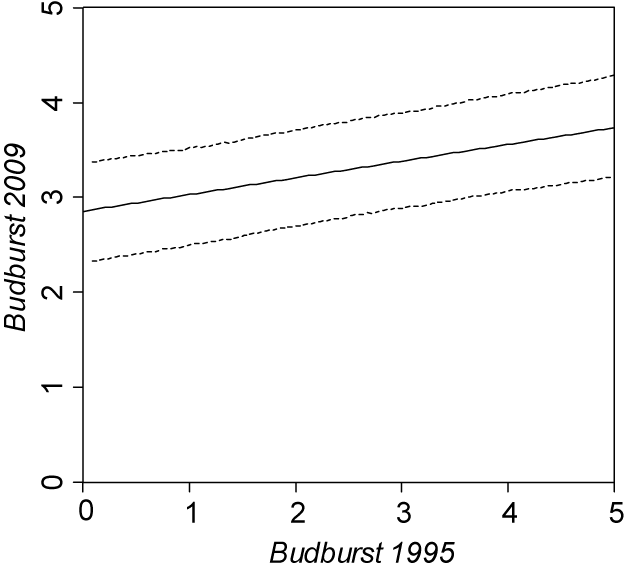
­

**Figure S4.1** Illustration of the estimated relationship (solid line) with 95% confidence intervals (dashed lines) between spring bud-burst phenology for the same 582 trees in 2009 and 1995. Estimates of the slope parameter and its confidence intervals are provided in Table S4.1. Axis units are on an ordinal scale from closed buds (0) to fully developed leaves (5 - scoring methodology is described in detail in supplementary file Data S1).

*References*

Askew, R. R. 1962. The Distribution of Galls of Neuroterus (Hym, Cynipidae) on Oak. Journal of Animal Ecology **31**:439-455.

Baayen, R. H. 2011. languageR: Data sets and functions with "Analyzing Linguistic Data: A practical introduction to statistics". R package version 1.2.

Bates, D., M. Maechler, and B. Bolker. 2011. lme4: Linear mixed-effects models using S4 classes. R package version 0.999375-39.

Bolker, B. M., M. E. Brooks, C. J. Clark, S. W. Geange, J. R. Poulsen, M. H. H. Stevens, and J. S. S. White. 2009. Generalized linear mixed models: a practical guide for ecology and evolution. Trends in Ecology & Evolution **24**:127-135.

Crawley, M. J. and M. Akhteruzzaman. 1988. Individual Variation in the Phenology of Oak Trees and Its Consequences for Herbivorous Insects. Functional Ecology **2**:409-415.

R Development Core Team. 2011. R: A language and environment for statistical computing. R Foundation for Statistical Computing, Vienna, Austria.

Sinclair F.H. (2012). Community level consequences of adaptive management through Climate Matching: oak galls as a model system. Unpublished Doctoral thesis, University of Edinburgh.
